# High Regnase-1 Expression Is Associated with an Immunosuppressive Tumor Microenvironment and Aggressive Features in Glioma Patients

**DOI:** 10.3390/cancers18101658

**Published:** 2026-05-20

**Authors:** Kenza Miyara, Hamza Benthami, Hayat Miftah, Saadia Ait Ssi, Chaimae Boulhen, Abdelhakim Lakhdar, Abdallah Badou

**Affiliations:** 1 Immuno-Genetics and Human Pathology Laboratory (LIGEP), Faculty of Medicine and Pharmacy, Hassan II University, Casablanca 20250, Morocco; kenza.miyara1-etu@etu.univh2c.ma (K.M.); hamza.benthami-etu@etu.univh2c.ma (H.B.); hayat.miftah-etu@etu.univh2c.ma (H.M.); saadia.aitssi-etu@etu.univh2c.ma (S.A.S.); chaimae.boulhen-etu@etu.univh2c.ma (C.B.); 2 Department of Neurosurgery, Ibn Rochd University Hospital Center (UHC), Casablanca 20360, Morocco; abdelhakim.lakhdar@univh2c.ma; 3 Laboratory of Research on Neurologic, Neurosensorial Diseases and Handicap, Faculty of Medicine and Pharmacy, Hassan II University, Casablanca 20250, Morocco; 4 City of Innovation and Technology Transfer (CITT), Hassan II University, Casablanca 20000, Morocco

**Keywords:** Regnase-1, tumor microenvironment, immunosuppression, immune checkpoint regulation, antitumor immune response, immunotherapy, glioma prognosis

## Abstract

**Background/Objectives**: Gliomas are among the most aggressive primary brain tumors in adults, characterized by profound molecular heterogeneity and poor response to conventional therapies. Immunotherapy has transformed outcomes in several cancers, yet glioma remains largely refractory, due in part to an immunosuppressive tumor microenvironment. Post-transcriptional regulation of gene expression is increasingly recognized as a key mechanism controlling immune cell function in tumors. Regnase-1, an endoribonuclease regulating the stability of inflammation- and immunity-related mRNAs, is a central modulator of immune responses; however, its role in glioma progression and immune modulation remains poorly understood. This study aimed to evaluate Regnase-1 expression in glioma and investigate its association with tumor grade, prognosis, and immune microenvironment characteristics. **Methods**: Regnase-1 transcript levels were evaluated by RT-PCR in tumor samples from 40 Moroccan glioma patients and validated using transcriptomic data from The Cancer Genome Atlas (TCGA, *n* = 672) and the Chinese Glioma Genome Atlas (CGGA, *n* = 959). Bioinformatic analyses and statistical assessments were performed using established pipelines. **Results**: Regnase-1 expression was significantly elevated in glioblastoma, IDH-wildtype tumors, and higher tumor grades, correlating with poorer overall survival, and emerging as an independent prognostic factor in the CGGA cohort. High Regnase-1 expression was associated with enrichment of pathways related to angiogenesis, hypoxia, invasion, and immune evasion. Tumors with elevated Regnase-1 showed reduced infiltration of effector immune cells (CD8^+^ T cells, Th1 cells) and increased presence of immunosuppressive populations, including regulatory T cells, myeloid-derived suppressor cells, and M2 macrophages. Single-cell analyses further highlighted exhausted CD8^+^ T cells and regulatory T cells as major populations linked to Regnase-1 expression. Notably, Regnase-1 expression also exhibited strong positive correlations with multiple inhibitory immune checkpoint pathways. **Conclusions**: Elevated Regnase-1 expression defines an aggressive, immunosuppressive glioma phenotype and is associated with poor prognosis, supporting its potential as a prognostic biomarker and a target for immunomodulatory strategies.

## Simple Summary

Gliomas are the most common malignant brain tumors in adults and remain associated with poor clinical outcomes despite advances in standard therapies. Although immunotherapy has transformed the treatment landscape of several malignancies, its efficacy in glioma remains limited, largely due to a highly heterogeneous and immunosuppressive tumor microenvironment that impairs effective antitumor immune responses. Regnase-1 is a key regulator of inflammation and immune signaling, acting through the control of messenger RNA stability. Increasing evidence highlights its role in shaping immune cell function; however, its relevance in glioma biology remains insufficiently explored. In this study, we investigated the expression of Regnase-1 in glioma patients and analyzed its association with the tumor immune landscape. Our findings indicate that Regnase-1 is linked to immunosuppressive features and may contribute to immune evasion, supporting its potential as a biomarker and a promising target for future immunotherapeutic strategies in glioma.

## 1. Introduction

Gliomas constitute the predominant form of primary malignant tumors of the central nervous system (CNS), with glioblastomas in particular posing a major therapeutic challenge [1]. Their diverse cellular composition, pronounced molecular heterogeneity, and highly infiltrative growth pattern drive unpredictable disease progression and constrain the effectiveness of current treatment approaches [2]. Despite advances in surgical management and combined chemo-radiotherapy, prognosis remains poor, particularly for patients with high-grade gliomas, as indicated by a median overall survival of 15–20.9 months [3,4,5,6,7]. Recurrent disease remains a defining feature of glioblastomas, highlighting the urgent need to further elucidate regulatory mechanisms involved in glioma progression [8].

Growing evidence identifies the glioma tumor microenvironment (TME) as a critical determinant governing tumor dynamics and therapeutic responses [9,10]. This ecosystem exhibits a profoundly immunosuppressive landscape marked by dysfunctional immune cell phenotypes and impaired pro-inflammatory signaling that collectively sustain tumor progression [11,12,13]. Indeed, the central role of the immune network within the TME underscores the therapeutic value of restoring effective antitumor immunity, as demonstrated by the success of immune checkpoint inhibition across multiple malignancies. Glioma, by contrast, exhibits limited therapeutic responsiveness, reflecting intricate immune evasion circuits that extend beyond classical therapeutic targets [14,15]. Post-transcriptional regulation represents a distinct layer of immune control in which RNA stability and degradation fine-tune cellular signaling and immune responses [16]. Emerging mechanistic insights indicate that oncogenesis reflects the interplay between genetic alterations and post-transcriptional RNA regulation [17].

In fact, RNA stability dynamics coordinate cytokine signaling, immune cell programming, and inflammatory equilibrium within the TME, positioning RNA-binding proteins and endoribonucleases as central orchestrators of tumor immunity [18,19]. Within this post-transcriptional regulatory axis, the Regnase family has emerged as a critical class of endoribonucleases that restrain immune responses by limiting inflammatory mediator expression [20]. Under physiological conditions, Regnase proteins operate to tightly regulate immune activation, maintaining homeostatic balance [20,21]. In states of regulatory imbalance, aberrant Regnase activity disrupts immune equilibrium, fostering microenvironmental signaling permissive to tumor progression [21,22,23,24,25,26,27].

The Regnase family comprises four endoribonucleases, Regnase-1 to -4, characterized by a conserved CCCH-type zinc finger domain coupled to a PilT N-terminal (PIN)-like RNase domain [28]. Zinc-dependent recognition of mRNAs encoding pro-inflammatory cytokines and immune regulators by the CCCH motif, together with PIN-domain catalytic activity, enables Regnase proteins to execute targeted transcript degradation [28,29,30]. By post-transcriptionally constraining gene expression, this mechanism stabilizes cytokine signaling and prevents persistent immune activation that may favor autoimmune reactions or cancer development [20]. In recent years, advances in Regnase biology have distinguished Regnase-1 as a central regulator of both innate and adaptive immune homeostasis with emerging therapeutic potential [31,32]. In fact, Regnase-1 maintains low constitutive expression across most human tissues with cytoplasmic localization, but is rapidly and strongly upregulated in response to inflammatory stimulation, particularly in immune effector cells, including macrophages, CD4^+^ and CD8^+^ T cells, B cells, dendritic cells and natural killer (NK) cells [33,34,35,36]. Mechanistically, by selectively degrading mRNAs encoding key cytokines and immune mediators, Regnase-1 regulates the intensity and duration of immune activation, thereby shaping T-cell function, macrophage polarization, and inflammatory dynamics [33]. Wei et al. reported a defining advance in understanding the role of Regnase-1 in cancer immunology, demonstrating that its CD8^+^ T cell-specific deletion in preclinical melanoma and leukemia models enhances T cell persistence, effector function, metabolic fitness, and antitumor activity, partly through BATF derepression, thereby sustaining robust antitumor responses [37]. Similarly, Sun et al. showed that Regnase-1 deletion in NK cells increases IFN-γ production, tumor infiltration, and antitumor response efficacy in melanoma mouse models through OCT2 upregulation [38]. Consistent with this rationale, glioma progression is shaped by impaired T-cell and NK-cell function and disrupted cytokine signaling, a pattern further reflected in the limited efficacy of classical immune checkpoint inhibition, thereby positioning the immunoregulatory properties of Regnase-1 as a compelling avenue for further investigation [11,12,39]. While Regnase-1 exerts tumor cell-intrinsic pro-oncogenic effects in glioma through VEGFA upregulation and ERK signaling, its immunological significance in this context remains largely unexplored [40]. This gap raises critical questions regarding the association of Regnase-1 expression with clinicopathological features, the immune microenvironment, and its role in glioma-associated immune suppression in patients.

To address this, we examined Regnase-1 expression patterns in fresh glioma biopsies from Moroccan patients, alongside two independent American and Chinese cohorts, the Cancer Genome Atlas (TCGA) and the Chinese Glioma Genome Atlas (CGGA). Our findings reveal a notable association between Regnase-1 transcripts and immunosuppressive features of the glioma microenvironment, suggesting that Regnase-1 may serve as a marker of immune evasion and a promising target for future immunotherapeutic approaches in glioma patients.

## 2. Materials and Methods

### 2.1. Patients and Samples

In this study, mRNA expression levels were evaluated in tumor samples from 40 glioma patients treated at the Ibn Rochd University Hospital Center from May 2016 to April 2022. Fresh biopsies were obtained immediately following surgical resection. Patients were enrolled based on predefined inclusion criteria, including a histopathologically confirmed diagnosis of invasive glioma, and provision of written informed consent. None of the patients received chemotherapy or radiotherapy prior to tumor resection. Exclusion criteria comprised absence of informed consent, failure to detect expression of the β-actin housekeeping gene, and tumors not diagnosed as glioma.

Relevant clinicopathological characteristics, including patient age, tumor grade, and IDH mutation status, were determined according to established diagnostic protocols per the 2016 World Health Organization (WHO) Classification of Tumors of the Central Nervous System, with data sourced from hospital records.

### 2.2. Total RNA Isolation and Reverse Transcription

Total RNA was isolated from 40 glioma biopsy specimens, processed either fresh or following frozen storage, using TRIzol reagent (Invitrogen, Massy, France), strictly following the manufacturer’s instructions. The purified RNA was resuspended in RNase-free water and either processed immediately for downstream applications or stored at −80 °C for later use. RNA concentration and purity were determined using a NanoVue™ Plus spectrophotometer (GE Healthcare, Hatfield, UK). For first-strand complementary DNA (cDNA) synthesis, reverse transcription was performed using 0.5 μg of total RNA per reaction to ensure comparability across samples. The 20-μL reaction mixture consisted of 1 μL Random Hexamer Primer (25 μg/mL; Bioline, Livron-sur-Drôme, France), 0.5 μL RNase inhibitor (Invitrogen, Massy, France), 0.5 μL Tetro Reverse Transcriptase (Bioline, Livron-sur-Drôme, France), 4 μL 5× Tetro Reverse Transcriptase buffer, 4 μL dNTP mix (10 mM each), and RNase-free water to volume. The thermal cycling conditions were as follows: primer annealing at 25 °C for 10 min, reverse transcription at 42 °C for 45 min, enzyme inactivation at 70 °C for 15 min, followed by holding at 4 °C.

### 2.3. Real-Time PCR Assay

Relative gene expression was quantified using real-time polymerase chain reaction (PCR) with SYBR™ Green PCR Master Mix (Thermo Fisher Scientific, Waltham, MA, USA) on the croBEE^®^ real-time PCR detection system. Gene-specific primers targeting Regnase-1 and the housekeeping gene β-actin were diluted from 100 µM stock solutions to 10 μM working concentrations prior to use. Each 20 μL reaction mixture contained 10 μL SYBR™ Green Master Mix, 7 μL RNase-free water, 0.5 μL forward primer, and 0.5 μL reverse primer. Reactions were initiated by the addition of 2 μL cDNA template. No-template controls (NTCs) were prepared by replacing the cDNA with 2 μL of RNase-free water.

Thermal cycling conditions consisted of an initial polymerase activation and template denaturation at 95 °C for 10 min, followed by 40 cycles of denaturation at 95 °C for 15 s and combined annealing/extension at 60 °C for 60 s. Amplification specificity was confirmed by melt curve analysis performed immediately after cycling. In cases where additional verification was required, PCR products were analyzed by 2% agarose gel electrophoresis to confirm amplicon size and specificity.

Cycle threshold (Ct) values were automatically determined from the fluorescence data collected during the extension step. A matched normal tissue calibrator could not be obtained owing to ethical constraints; relative gene expression was therefore calculated using the 2^−ΔCt^ method with β-actin as the housekeeping gene. All primer sequences used in this study are listed in Table 1.

### 2.4. Public Datasets Acquisition and Preprocessing

Complementing our in-house cohort analysis, the computational study integrated transcriptomic and clinicopathological data from publicly available American and Chinese glioma patient cohorts, sourced, respectively, from the TCGA and CGGA datasets.

For TCGA patients, gene expression data were downloaded from the UCSC Xena platform (https://xenabrowser.net/) for the Lower Grade Glioma (LGG) and Glioblastoma (GBM) cohorts. Specifically, gene expression Illumina RNA-seq matrices corresponding to the GDC TCGA LGG cohort and the GDC TCGA GBM cohort were retrieved in STAR-aligned transcript-per-million (TPM) normalization format with log2(x + 1) transformation. To ensure consistent gene annotation, gene identifiers were standardized and mapped using the HGNC-GENCODE v24 (hg19) reference annotation to maintain consistent gene labeling across datasets. Corresponding clinicopathological data for TCGA samples were retrieved from the cBioPortal platform (https://www.cbioportal.org/). Clinical records were integrated with gene expression matrices through paired unique patient/sample identifiers, allowing direct correspondence between RNA-seq and clinicopathological data. Only cases presenting matched transcriptomic–clinicopathological profile data were retained for downstream analysis. Samples with missing or incomplete records were excluded. TPM gene expression data from both LGG and GBM patients were merged, resulting in a global TCGA cohort of *n* = 672.

Independent validation datasets were obtained from the CGGA cohort and downloaded from the CGGA portal (http://www.cgga.org.cn/), including RNA-seq batch 1 (mRNAseq_693) and batch 2 (mRNAseq_325). These datasets were processed using the same inclusion criteria, retaining only samples with matched gene expression and clinical profiles to ensure analytical consistency. Of note, clinicopathological data in the TCGA and CGGA datasets reflect classifications assigned according to the WHO Classification of Tumors of the Central Nervous System in use at the time of data collection by the contributing institutions.

To minimize non-biological technical variability introduced by batch differences, batch correction was performed on the log2(FPKM + 1) transformed expression matrix using removeBatchEffect from the limma package [41], applying an intercept-only model. Prior to correction, patients were screened for statistical outliers in a data-driven manner using principal component analysis (PCA), followed by Mahalanobis distance estimation in principal component space [42]. Samples exceeding the predefined multivariate threshold were excluded from subsequent analyses (Supplementary Figure S1).

To rigorously assess the initial technical batch effect, permutational multivariate analysis of variance (PERMANOVA) was performed on the expression data using the vegan R package (999 permutations) [43,44], with effect size (R^2^) and associated *p*-values used to quantify variance attributable to batch factors. The efficacy of the batch correction procedure was evaluated by repeating PERMANOVA on the adjusted datasets, confirming that batch-associated effects were effectively corrected while biologically relevant variables were preserved (Supplementary Figure S1). Following correction, log2(FPKM + 1) expression values were back-transformed to the linear scale, constrained to a minimum expression floor to prevent negative or near-zero artifacts, and converted from FPKM to TPM on a per-sample basis to ensure cross-sample comparability by scaling each gene’s expression by the sample-wise sum and multiplying by 10^6^. The final normalized outputs consisted of TPM matrices and log2(TPM + 1) matrices for downstream integrative analyses, comprising a total of *n* = 959 samples following preprocessing and quality control. All preprocessing steps and corresponding statistical analyses were conducted using RStudio software (v2025.05.1+513).

### 2.5. Multivariate Cox Proportional Hazards Survival Analysis

To assess the independent prognostic value of Regnase-1 gene expression in our cohorts, multivariate Cox proportional hazards regression was performed in RStudio using the survival, survminer, tidyverse, and mice packages [45,46,47,48,49], integrating clinicopathological covariates with gene expression status. Clinical data were curated using the tidyverse suite to standardize sample identifiers, convert survival time and event status variables, and retain cases with non-missing overall survival data. Gene expression values were dichotomized into high- and low-expression groups using a cohort-specific median threshold for Regnase-1, with the high-expression group modeled relative to the low-expression reference category. Missing values in clinicopathological covariates were addressed using multiple imputation by chained equations (MICE; 10 imputations) with a predictor matrix derived from covariate correlation patterns (Supplementary Figure S2). Clinicopathological variables demonstrating independent prognostic significance within their respective cohorts were retained as adjustment covariates when evaluating the independent prognostic effect of Regnase-1. Hazard ratios (HRs) and corresponding measures of statistical significance were reported. Forest plots were generated using survminer.

### 2.6. Gene Set Enrichment Analysis

Gene Set Enrichment Analysis (GSEA) was performed to identify coordinated pathway-level transcriptional activity by testing whether predefined biological gene sets were non-randomly enriched across a ranked gene expression profile, comparing tumors exhibiting high versus low Regnase-1 expression phenotypes defined using the cohort-specific median expression threshold. GSEA was conducted using GSEA software (version 4.3.2) [50].

Gene sets were sourced from the Hallmark, Curated, and Gene Ontology (GO) collections in the Molecular Signatures Database (MSigDB), accessed via the GSEA/MSigDB portal https://www.gsea-msigdb.org/gsea/downloads.jsp (accessed on 15 June 2025). Selected gene sets were subsequently organized into biologically interpretable categories to facilitate hypothesis-driven pathway evaluation. Log-transformed expression values were used as GSEA input, calculated as log2(TPM + 1) to stabilize variance and support cross-sample comparability for GSEA. Statistical significance was assessed through 1000 gene set permutations to derive nominal *p*-values, with enriched pathways considered significant at nominal *p* < 0.05 and a false discovery rate (FDR) q-value < 0.25. Normalized enrichment scores (NES), nominal *p*-values, FDR q-values, and the full set of enriched gene signatures are provided in Supplementary Tables S1 and S2.

### 2.7. Estimation of Tumor-Infiltrating Immune Cell Abundance

Tumor microenvironment (TME) immune cell infiltration was estimated using the ImmunoCellAI R package (v2022) [51], applying the RNA-seq module to TPM-normalized gene expression data to infer the relative abundance of 24 immune cell types. Complementary immune deconvolution was performed using the deconvo_tme function from the IOBR package (v1.0) [52], which provided both CIBERSORT-derived cell abundance scores and TIMER estimates with the LGG and GBM criteria specified per patient. In addition, xCell was applied using the xCellAnalysis function from the xCell R package [53]. Spearman correlations between Regnase-1 expression and the immune-cell fractions derived from these tools were visualized with heatmaps generated using the pheatmap R package.

In addition, immune-related signatures for tumor-associated macrophages (TAMs), cancer-associated fibroblasts (CAFs), and myeloid-derived suppressor cells (MDSCs) were generated using the z-score method implemented within IOBR package. Resulting infiltration fractions were subsequently stratified according to Regnase-1 expression status, classifying samples into previously defined high- and low-expression groups.

### 2.8. Immune Cell Gene Expression Profiling via Digital Cytometry

To characterize immune-related gene expression within defined immune cell populations, we applied the CIBERSORTx digital cytometry tool [54]. Pseudo-bulk gene expression profiles (GEP) for CD8^+^ T cells, regulatory T cells (Tregs), natural killer (NK) cells, and M2 macrophages were computationally inferred from TPM-normalized bulk RNA-seq tumor transcriptomes. Analyses were executed locally within a Docker container environment using Docker Desktop (v4.24.0), employing the LM22 signature matrix [54] as the deconvolution reference. Consistent with input requirements, TPM expression values were provided in linear space prior to stratification by Regnase-1 expression.

### 2.9. Immune Functional Score Analysis

To examine immune-related functional states across the defined patient subgroups, two complementary scoring algorithms were applied, namely the ESTIMATE approach [55] and gene signature-based z-scoring. The ESTIMATE method was first used to compute stromal, immune, and tumor purity scores from bulk RNA-seq expression profiles. Next, gene signature z-score analysis was performed to assess signatures of T-cell exhaustion, cellular senescence, T-cell anergy, and immune evasion between Regnase-1 groups. All computations were conducted using the IOBR R package using RStudio.

### 2.10. Tumor Immune Single-Cell Transcriptomic Profiling

To further define the oncoimmune transcriptional patterns at the cellular level, we queried the Tumor Immune Single-cell Hub 2 (TISCH2), a curated scRNA-seq database that provides uniformly processed and pre-annotated data from human tumors [56]. From this repository, we accessed two glioma datasets, Glioma_GSE163108_10X and Glioma_GSE131928_10X, to examine the distribution of Regnase-1 expression across distinct pre-annotated cell populations, including malignant cells, stromal cells, and diverse tumor-infiltrating immune subsets.

### 2.11. Statistical Analysis and Data Visualization

Statistical analyses and graphical representations were performed using GraphPad Prism (v10.2.3) and RStudio. Data normality was assessed using the Shapiro–Wilk and Kolmogorov–Smirnov tests. Comparisons between two independent groups were performed using the Mann–Whitney U test, whereas differences among more than two groups were assessed using the Kruskal–Wallis test with appropriate post hoc pairwise comparisons. Welch’s *t*-test was applied when comparing mean expression values between two groups. Correlations between continuous variables were assessed using Spearman’s rank correlation coefficient. The prognostic value of Regnase-1 expression was investigated by Kaplan–Meier survival analysis, with differences in overall survival between groups compared using the log-rank (Mantel–Cox) test. Statistical significance was defined as a two-sided *p*-value < 0.05 after Benjamini–Hochberg correction (FDR < 0.05).

### 2.12. Ethics Approval and Consent to Participate

This study was conducted in accordance with the ethical principles outlined in the Declaration of Helsinki. Approval was granted by the Ethics Committee for Biomedical Research (CERB) of Ibn Rochd University Hospital Center, Casablanca, Morocco (28/15).

Written informed consent was obtained from all participants prior to inclusion. For individuals unable to provide consent due to a critical medical condition, informed consent was obtained from a parent, legal guardian, or legally authorized representative in accordance with institutional requirements. Before providing consent, all participants or their legal representatives were informed about the study objectives and procedures. All study procedures were performed in compliance with applicable national and international guidelines and regulations.

## 3. Results

### 3.1. Regnase-1 Expression Was Associated with Aggressive Clinicopathological Features and Poor Prognosis in Patients with Invasive Glioma

To investigate the expression pattern of Regnase-1 and assess its potential contribution to glioma development, we analyzed an in-house cohort comprising 40 patients with invasive gliomas (Table 2). Regnase-1 mRNA levels were measured by RT-PCR and normalized to β-actin expression.

**Figure 1:**
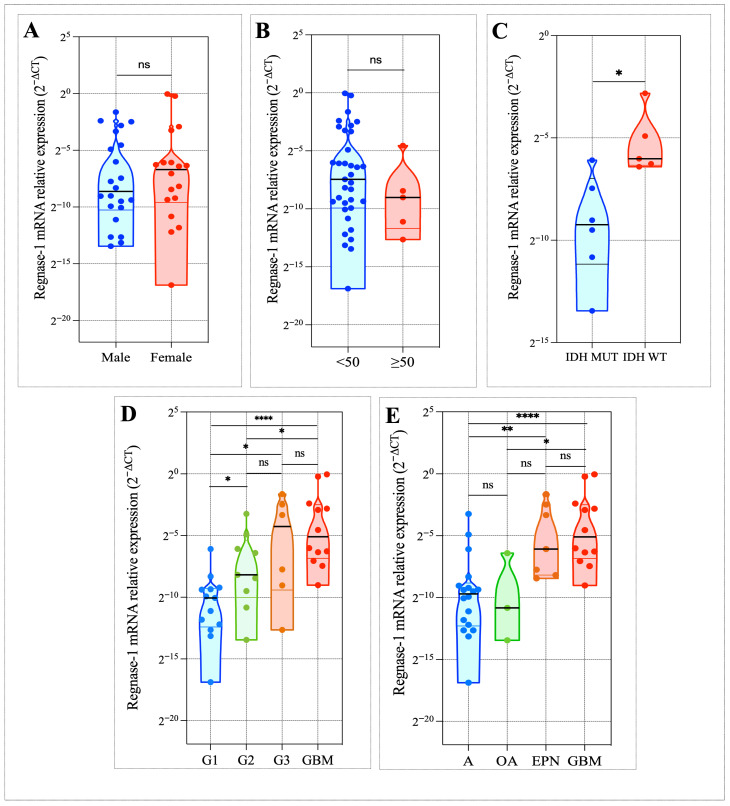
Elevated Regnase-1 expression was associated with aggressive clinicopathological features in our Moroccan glioma cohort. Regnase-1 mRNA levels were quantified in 40 invasive glioma samples by RT-PCR, normalized to β-actin, and presented as 2^−ΔCt^ values, with expression displayed on a log_2_-scaled axis. (**A**,**B**) No significant association was identified between Regnase-1 expression and patient age (*p* = 0.3370) or sex (*p* = 0.5999). (**C**) A marked upregulation of Regnase-1 expression was observed in IDH-wildtype gliomas compared with IDH-mutant tumors (*p* = 0.0173). (**D**) Regnase-1 expression levels were significantly increased in grade IV gliomas relative to grade I tumors (*p* = 0.0005). (**E**) High Regnase-1 expression was observed in the glioblastoma (GBM) subtype (*p* = 0.0002). Significance was defined as Benjamini–Hochberg corrected FDR < 0.05. * *p* < 0.05, ** *p* < 0.01, **** *p* < 0.0001, and ‘ns’ for no significant difference.

To explore the clinical relevance of Regnase-1, we next examined its association with major clinicopathological parameters, including patient age, sex, IDH mutation status, tumor grade, and histological subtype. Regnase-1 expression showed no significant association with patient age (*p* = 0.3370) (Figure 1A) or sex (*p* = 0.5999) (Figure 1B). However, Regnase-1 expression was significantly elevated in clinically aggressive glioma phenotypes. IDH-wildtype gliomas exhibited higher Regnase-1 transcript levels compared with IDH-mutant tumors (*p* = 0.0173) (Figure 1C). A similar pattern was observed across tumor grades, with grade IV gliomas showing significantly increased expression relative to grade I tumors (*p* = 0.0005) (Figure 1D). Consistent with these findings, expression levels were markedly elevated in the glioblastoma (GBM) subtype (*p* = 0.0002) (Figure 1E).

Taken together, these findings indicate that elevated Regnase-1 expression is associated with aggressive clinicopathological features in our in-house cohort.

**Figure 2:**
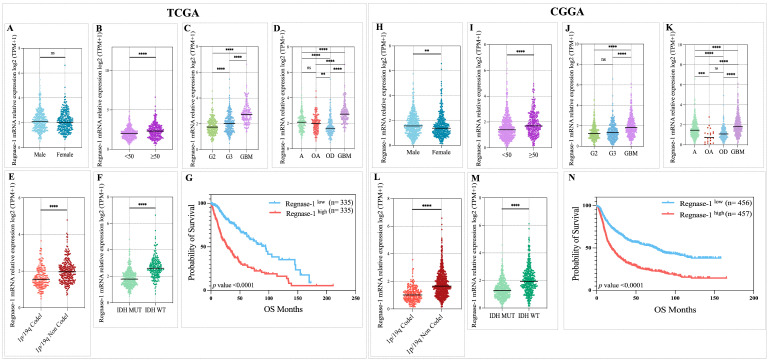
Higher Regnase-1 transcript levels were associated with adverse clinicopathological features and poor prognosis in the TCGA (*n* = 672) and CGGA (*n* = 959) glioma cohorts. (**A**) No significant association was identified between Regnase-1 expression and sex in the TCGA cohort (*p* = 0.2803). (**H**) Elevated Regnase-1 expression was observed in male glioma patients within the CGGA cohort (*p* = 0.0043). (**B**,**I**) Regnase-1 expression was significantly increased in glioma patients aged ≥50 years in both TCGA (*p* < 0.0001) and CGGA (*p* < 0.0001). (**C**,**J**) Regnase-1 expression levels were significantly higher in grade IV gliomas compared with grade I tumors in TCGA (*p* < 0.0001) and CGGA (*p* < 0.0001). (**D**,**K**) Regnase-1 gene expression was significantly elevated in the aggressive glioblastoma (GBM) subtype in both cohorts (*p* < 0.0001). (**E**,**L**) Regnase-1 expression was markedly higher in 1p/19q non-codeleted gliomas than in 1p/19q codeleted tumors in TCGA (*p* < 0.0001) and CGGA (*p* < 0.0001). (**F**,**M**) Increased Regnase-1 transcript levels were observed in IDH-wildtype gliomas compared to IDH-mutant cases in both cohorts (*p* <0.0001). (**G**,**N**) Glioma patients with high Regnase-1 expression exhibited poorer overall survival compared with the low-expression group based on Kaplan–Meier analysis in TCGA and CGGA cohorts. Blue and red curves represent patients with low and high Regnase-1 expression, respectively. Significance was defined as Benjamini–Hochberg corrected FDR < 0.05. ** *p* < 0.01, **** *p* < 0.0001, and ‘ns’ for no significant difference.

To corroborate our initial observations, we extended the assessment of the clinical significance of Regnase-1 by analyzing large-scale transcriptomic datasets from the TCGA and CGGA cohorts, encompassing a total of 672 and 959 glioma patients, respectively (Table 3 and Table 4). Across both cohorts, higher Regnase-1 transcript levels were consistently linked to clinicopathological features indicative of aggressive glioma biology. Of note, no significant association with sex was observed in TCGA, whereas male patients in the CGGA cohort exhibited modestly higher expression levels (Figure 2A,H). Age-related comparisons further revealed significantly increased expression in patients aged ≥50 years across TCGA and CGGA cohorts (Figure 2B,I).

Marked Regnase-1 upregulation was observed in grade IV gliomas compared with grade I tumors (Figure 2C,J), and expression levels were similarly elevated in the highly aggressive glioblastoma (GBM) subtype (Figure 2D,K). Furthermore, gliomas lacking the 1p/19q codeletion displayed substantially higher Regnase-1 expression than codeleted tumors (Figure 2E,L). Consistent with these patterns, IDH-wildtype gliomas exhibited a pronounced increase in Regnase-1 expression relative to IDH-mutant cases (Figure 2F,M). Survival analyses supported the prognostic relevance of Regnase-1, showing that patients with high expression had significantly reduced overall survival in both TCGA and CGGA datasets (Figure 2G,N). Collectively, these findings independently corroborate that elevated Regnase-1 is strongly associated with advanced disease features and poor prognosis in glioma.

**Figure 3:**
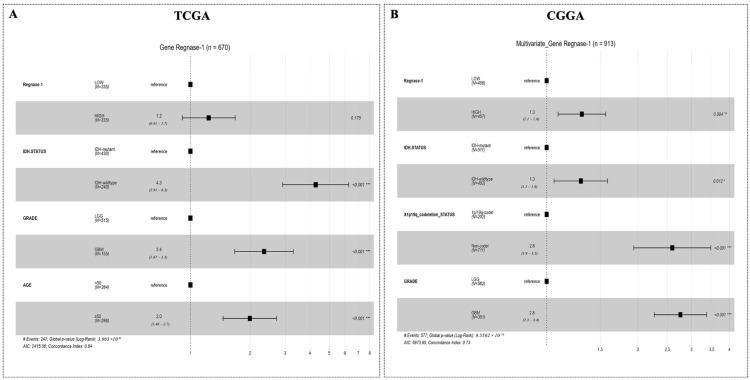
Multivariable cox proportional hazards analyses of Regnase-1 expression and cohort-specific prognostic factors. Forest plots display adjusted hazard ratios for overall survival derived from multivariable models in the TCGA (**A**) and CGGA (**B**) cohorts. Points represent hazard ratio estimates and horizontal lines indicate 95% confidence intervals. Statistical significance is denoted as follows: * *p* < 0.05, ** *p* < 0.01, *** *p* < 0.001. Significance was defined as Benjamini–Hochberg corrected FDR < 0.05.

To further characterize the prognostic value of Regnase-1, multivariate Cox proportional hazards regression analyses were performed independently in the TCGA (Figure 3A) and CGGA (Figure 3B) glioma cohorts, adjusting for established clinicopathological variables.

In the TCGA cohort, higher Regnase-1 expression was associated with a trend toward increased hazard of death. However, this association did not reach statistical significance after multivariable adjustment for cohort-specific clinicopathological prognostic factors.

Strikingly, in the CGGA cohort, high Regnase-1 expression was significantly associated with increased hazard of death and remained an independent prognostic factor after adjustment for molecular features and tumor grade.

Overall, these findings suggest that Regnase-1 expression is associated with poor prognosis, though its utility as an independent biomarker may vary depending on the clinical and demographic composition of the cohort.

### 3.2. Regnase-1 Upregulation Was Associated with Enrichment of Oncogenic and Aggressive Pathway Signatures in Glioma Patients

**Figure 4:**
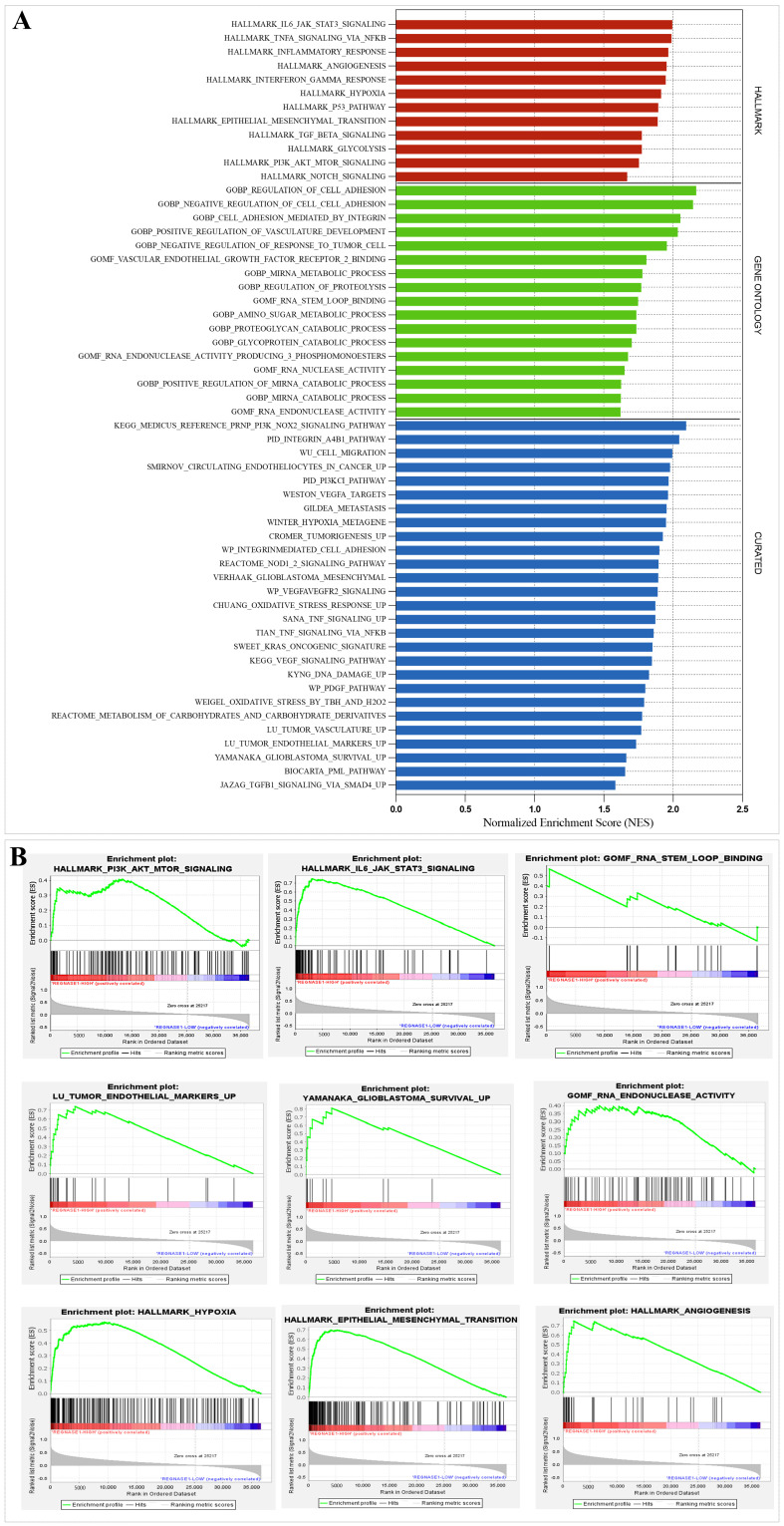
High Regnase-1 expression was associated with key signaling pathways and biological processes implicated in glioma pathogenesis and RNA regulatory mechanisms, as revealed by Gene Set Enrichment Analysis (GSEA). (**A**) Enrichment analysis plots illustrate significantly enriched gene sets in the Regnase-1-high phenotype, highlighting pathways and biological processes relevant to tumor progression, immune modulation, and post-transcriptional RNA regulation in the TCGA cohort. Bar colors indicate molecular signatures database categories: red represents Hallmark, green represents Gene Ontology, and blue represents Curated gene sets. (**B**) Representative enrichment plots illustrate positively enriched gene sets in the Regnase-1-high phenotype, reflecting the association between Regnase-1 expression and glioma aggressiveness in the TCGA cohort. Gene sets were considered significantly enriched at nominal *p* < 0.05 and FDR q-value < 0.25.

We conducted gene set enrichment analysis (GSEA) using predefined molecular signature collections from the Hallmark, Curated, and Gene Ontology human databases to evaluate functional pathways driving glioma proliferation, invasion, angiogenesis, and metastasis behavior (Figure 4). Strikingly, Regnase-1-high phenotype exhibited significant enrichment of pathways driving tumor progression including invasion, angiogenesis, hypoxia, epithelial–mesenchymal transition (EMT), and mesenchymal/endothelial glioblastoma phenotypes. Canonical oncogenic signaling pathways such as IL6–JAK–STAT3, TNF-α/NF-κB, TGF-β, and PI3K/AKT/mTOR were also significantly overrepresented. Notably, gene signatures related to mRNA degradation processes encompassing RNA nuclease and endonuclease activity, stem-loop RNA binding, and miRNA catabolism were enriched as well across TCGA and CGGA cohorts (Figure 4A,B; Supplementary Figure S3), suggesting that the endoribonuclease activity of Regnase-1 is associated with dynamic transcriptomic remodeling linked to tumor progression signatures.

### 3.3. Elevated Regnase-1 Expression Was Associated with Heterogeneous and Immune-Associated Features in the Glioma Microenvironment

**Figure 5:**
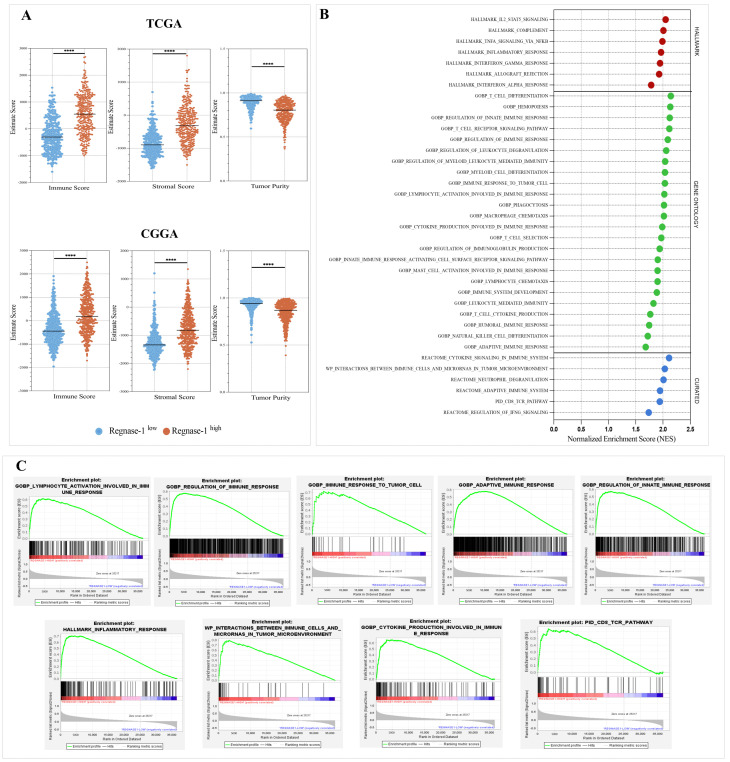
High Regnase-1 expression was associated with tumor microenvironment heterogeneity and immune-related pathways enrichment in glioma patients. Scores were determined using ESTIMATE and GSEA analyses. (**A**) High Regnase-1 tumors exhibited reduced tumor purity and elevated immune and stromal scores across both TCGA and CGGA cohorts. (**B**,**C**) Enrichment analysis plots illustrate significant enrichment of key immune-related biological processes in Regnase-1-high tumors from the TGGA cohort. For GSEA, gene sets were considered significantly enriched at nominal *p* < 0.05 and FDR < 0.25. Bubble colors indicate molecular signatures database categories: red represents Hallmark, green represents Gene Ontology, and blue represents Curated gene sets. For all other analyses, significance was defined as Benjamini–Hochberg corrected FDR < 0.05. **** *p* < 0.0001.

Given the aggressive molecular features associated with high Regnase-1 gliomas, we next sought to characterize their immune microenvironment. To this end, tumor purity and immune landscape features were assessed using ESTIMATE and GSEA approaches. Across the TCGA and CGGA cohorts, elevated Regnase-1 expression was consistently associated with an immune-enriched and compositionally diverse tumor microenvironment. This was reflected by increased immune and stromal scores, indicative of reduced tumor purity and greater microenvironmental heterogeneity (Figure 5A). Furthermore, analysis of high Regnase-1 gliomas revealed significant enrichment of immune-related pathways in TCGA and CGGA cohorts (Figure 5B,C; Supplementary Figure S4). Pathways associated with T cell activation and differentiation, cytokine signaling, and myeloid cell recruitment such as macrophage chemotaxis, leukocyte-mediated immunity were transcriptionally enriched, together with signatures related to NK cell differentiation. Collectively, these findings suggest that elevated Regnase-1 expression is associated with immune-related features characteristic of a dynamic glioma microenvironment.

### 3.4. High Regnase-1 Expression Was Associated with an Immunosuppressive and Tumor-Permissive Glioma Microenvironment

**Figure 6:**
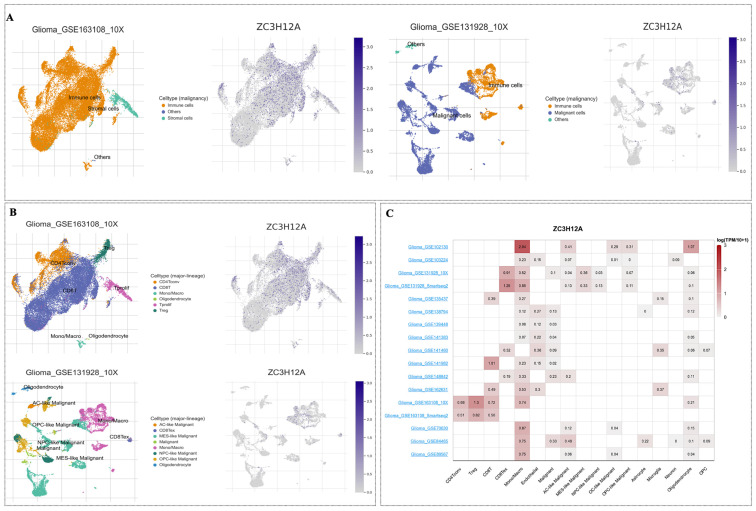
Regnase-1 gene expression in the glioma microenvironment at single-cell resolution. Analysis performed using the Tumor Immune Single-cell Hub 2 (TISCH2) scRNA-seq database. (**A**,**B**) Uniform Manifold Approximation and Projection (UMAP) visualization of two annotated glioma single-cell datasets, Glioma_GSE163108_10X and Glioma_GSE131928_10X, showing cellular landscapes and Regnase-1 expression across malignancy and major lineage populations. (**C**) Heatmap illustrating Regnase-1 expression patterns across different immune cell population in multiple RNA-seq datasets.

In light of the enriched immune pathway activity observed in Regnase-1-high gliomas, and recognizing the established role of immune infiltration in glioma progression, we next examined the cellular-level expression of Regnase-1 within the glioma tumor microenvironment using the Tumor Immune Single-cell Hub 2 (TISCH2) database. Two independent glioma single-cell RNA-seq datasets were analyzed, namely Glioma_GSE163108_10X and Glioma_GSE131928_10X (Figure 6A,B). Interestingly, Regnase-1 expression was detected in both immune cells and malignant/stromal cells (Figure 6A; Supplementary Figure S5).

Specifically, Regnase-1 is prominently expressed within immune cell populations, with the highest levels observed in Tregs and CD8^+^ T cells with an exhausted phenotype (Figure 6B). This distribution was further supported by similar patterns across immune subsets in additional TISCH2 cohorts (Figure 6C).

**Figure 7:**
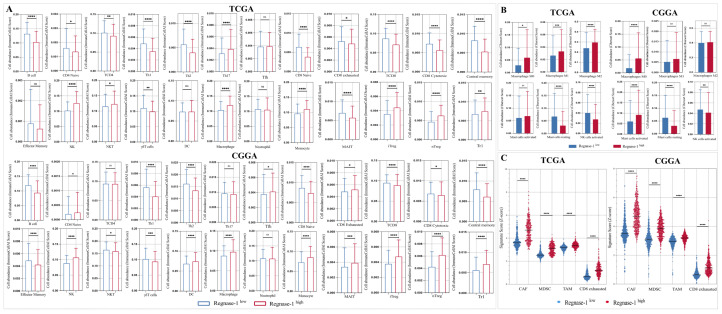
Immune infiltration profile according to Regnase-1 expression in glioma patients in the TCGA and CGGA cohorts. Immune cell fraction distributions were evaluated using the ImmunocellAI algorithm, Cibersort and Z-score signatures. (**A**) Immune cell composition within the glioma tumor microenvironment was assessed using ImmunoCellAI (v2022) across the TCGA and CGGA cohorts. (**B**) Distribution of complementary immune cell subsets analyzed using CIBERSORT in glioma samples from the TCGA and CGGA cohorts. (**C**) Z-score-based analysis was performed to assess the distribution of CAF, MDSC, TAM and CD8-exhausted in glioma samples from the TCGA and CGGA datasets. * *p* < 0.05, ** *p* < 0.01, *** *p* < 0.001, **** *p* < 0.0001, and “ns” for no significant difference. Significance was defined as Benjamini–Hochberg corrected FDR < 0.05.

Having established Regnase-1 expression within glioma-infiltrating immune cells at single-cell resolution, we next investigated whether its expression levels correlate with immune cell composition and dynamics in TCGA and CGGA glioma cohorts. Immune cell abundance was primarily estimated using the ImmuCellAI algorithm, assessing 24 distinct immune cell types, with complementary analyses performed using CIBERSORT and Z-score based signatures. In the TCGA cohort, patients with elevated Regnase-1 expression exhibited a significant reduction in key effector populations, including B cells, naïve CD4 T cells, CD4 T cells, Th1 cells, naïve CD8 T cells, cytotoxic CD8 T cells, central memory T cells, MAIT cells, and γδ T cells. In contrast, a marked increase in Th17 cells, NK cells, NKT cells, macrophages, monocytes, and regulatory T cell subsets, including Tr1, nTreg, and induced Tregs (iTregs), was observed (Figure 7A).

To further dissect the phenotypic states of NK cells, macrophages, and mast cells within the tumor microenvironment, CIBERSORT was employed to distinguish activated versus resting populations. High Regnase-1 gliomas demonstrated a significant infiltration of macrophage subsets (M0, M1, M2) and a reduction in resting mast cells and activated NK cells (Figure 7B). Moreover, elevated Regnase-1 expression was positively associated with increased infiltration of myeloid-derived suppressor cells (MDSCs), tumor-associated macrophages (TAMs), cancer-associated fibroblasts (CAFs), and exhausted CD8^+^ T cells, collectively suggesting a shift toward a more immunosuppressive microenvironment (Figure 7C).

Similar patterns were observed in the CGGA cohort, although with some cohort-specific distinctions. Tumors with high Regnase-1 expression exhibited diminished infiltration of anti-tumor effector populations, including B cells, Th1 cells, naïve CD8^+^ T cells, total CD8^+^ T cells, cytotoxic CD8^+^ T cells, central memory and effector memory T cells, and γδ T cells, while displaying enriched infiltration of pro-tumorigenic populations such as Treg subsets (Tr1, nTreg, iTreg), MDSCs, TAMs, CAFs, and exhausted CD8^+^ T cells (Figure 7A,C). Conversely, an increase in NK cells and CD8^+^ T cells was also detected. CIBERSORT analyses further revealed expanded macrophage subsets (M0, M2) and activated mast cells, accompanied by reduced resting mast cells and activated NK cells in Regnase-1-high tumors (Figure 7B).

These observations were further supported by correlation analyses in the same cohorts, revealing significant associations between Regnase-1 expression and protumoral immune cell populations (Supplementary Figure S6).

Consistent with these findings, supplementary analyses using TIMER indicated that higher Regnase-1 expression is associated with increased immune cell infiltration, while xCell analysis demonstrated significant correlations between Regnase-1 expression and multiple immune and stromal cell scores across TCGA and CGGA cohorts (Supplementary Figure S7).

Collectively, these results indicate that high Regnase-1 expression is associated with a pronounced shift toward an immunosuppressive tumor microenvironment, characterized by reduced infiltration of anti-tumor effector cells and increased abundance of pro-tumor immune and stromal populations.

**Figure 8:**
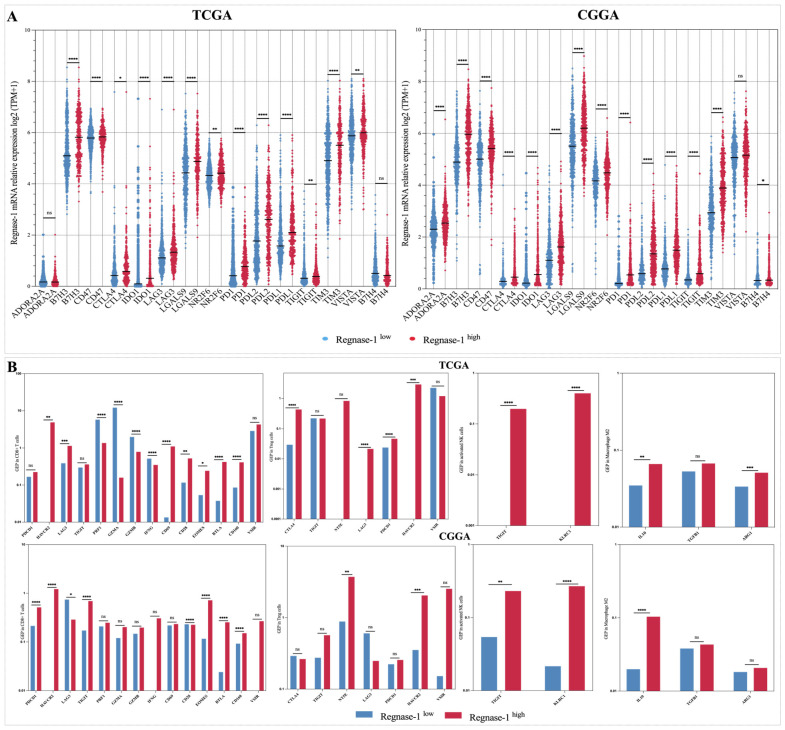
Regnase-1 overexpression was associated with the upregulation of inhibitory immune checkpoints and pro-tumoral chemokines in TCGA and CGGA cohorts. (**A**) An upregulation of inhibitory immune checkpoints was observed in glioma patients exhibiting high Regnase-1 expression. (**B**) Cell-type-specific expression of exhaustion and inhibitory markers in Regnase-1-high versus low gliomas using digital cytometry profiling in TCGA and CGGA. Welch’s *t*-test was used to compare mean gene expression profiles across groups, accounting for standard deviations and sample sizes. * *p* < 0.05, ** *p* < 0.01, *** *p* < 0.001, **** *p* < 0.0001, and ‘ns’ for no significant difference. Significance was defined as Benjamini–Hochberg corrected FDR < 0.05.

Having characterized the cellular landscape associated with Regnase-1, we next sought to define the molecular features underpinning its distinct immunosuppressive profile, with a focus on inhibitory immune checkpoints and protumoral mediators implicated in glioma immune escape. To this end, we examined a comprehensive panel of immune-modulatory molecules across the TCGA and CGGA cohorts. In the TCGA dataset, patients with high Regnase-1 expression displayed a consistent upregulation of multiple inhibitory immune checkpoints, including B7-H3, CD47, CTLA-4, IDO1, LAG-3, LGALS9, NR2F6, PD-1, PD-L1, PD-L2, TIGIT, TIM-3, VISTA, and B7-H4, whereas ADORA2A did not demonstrate a significant association (Figure 8A). A comparable pattern was observed in the CGGA cohort, with the notable exception that ADORA2A also exhibited a significant positive association with Regnase-1 expression. Correlation analyses in both cohorts further substantiated these findings, confirming robust associations between Regnase-1 and the examined inhibitory checkpoints (Supplementary Figure S8).

Following bulk immune profiling and recognizing the limitations of bulk transcriptomic approaches in capturing tumor cellular diversity, we used digital cytometry to characterize the molecular profiles of CD8^+^ T cells, M2-like macrophages, regulatory T cells (Tregs), and activated NK cells in TCGA and CGGA cohorts.

In Regnase-1-high tumors, CD8^+^ T cells displayed reduced expression of key effector and cytotoxic molecules, including IFNG, GZMA, GZMB, and PRF1 in TCGA patients, whereas similar trends in the CGGA cohort did not reach statistical significance.

Conversely, a broad panel of inhibitory and exhaustion-associated molecules was significantly upregulated, notably PDCD1 (PD-1), CD69, CD28, LAG3, HAVCR2 (TIM-3), TIGIT, BTLA, EOMES, and CD160, consistent with a dysfunctional and exhaustion-associated CD8^+^ T-cell phenotype. Tregs displayed elevated expression of multiple suppressive signature genes, including CTLA4, LAG3, PDCD1, HAVCR2, and NT5E, supporting heightened tolerogenic capacity. Conversely, expression of TIGIT and VSIR did not show significant differences between the two Regnase-1 groups.

M2-like macrophages in Regnase-1-high tumors showed significantly increased expression of canonical M2 polarization markers, including ARG1 and IL10, indicating enhanced pro-tumorigenic and anti-inflammatory activity in both TCGA and CGGA cohorts. No statistically significant differences in TGFB1 expression were observed in either cohort. Activated NK cells demonstrated markedly increased expression of inhibitory receptors, particularly TIGIT and KLRC1, suggesting impaired innate cytotoxic function and reduced activation status in Regnase-1-high gliomas (Figure 8B).

Together, these results support an association between high Regnase-1 expression and an exhaustion- and suppression-dominant immune phenotype in glioma.

**Figure 9:**
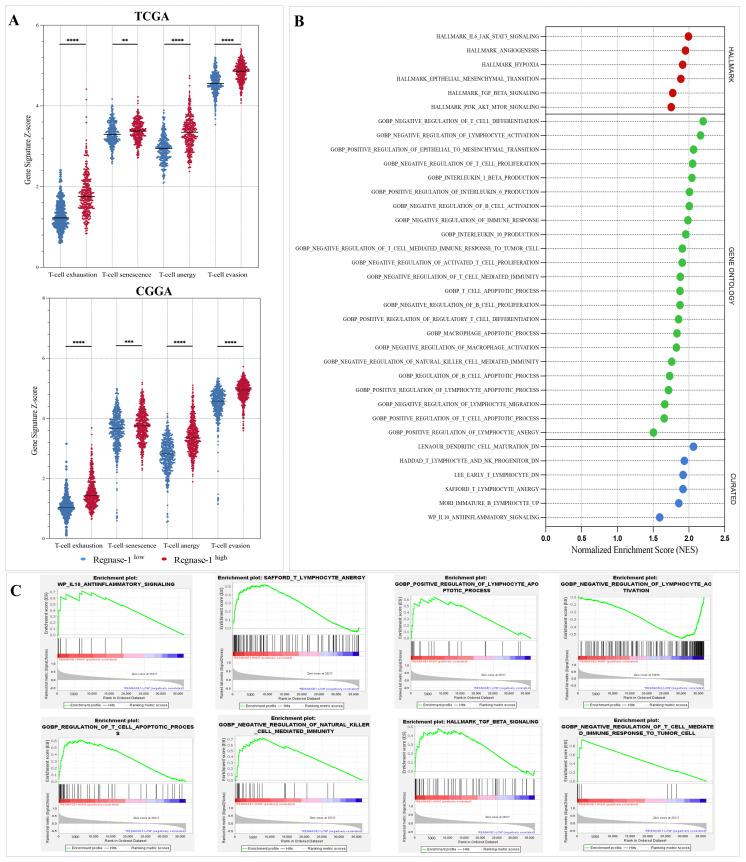
Elevated Regnase-1 expression is associated with impaired antitumor immune response signatures within the tumor microenvironment. Z-score and GSEA analyses were employed. (**A**) Regnase-1 overexpression is associated with increased T-cell dysfunction and immune escape signatures. (**B**,**C**) Enrichment analysis plots illustrate significant enrichment of immunosuppressive and tumor escape pathways in Regnase-1-high patients from the TGGA cohort. For GSEA, gene sets were considered significantly enriched at nominal *p* < 0.05 and FDR q < 0.25. Bubble colors indicate molecular signatures database categories: red represents Hallmark, green represents Gene Ontology, and blue represents Curated gene sets. For all other analyses, significance was defined as Benjamini–Hochberg corrected FDR < 0.05. ** *p* < 0.01, *** *p* < 0.001, **** *p* < 0.0001.

To deepen our investigation into the immunosuppressive role of Regnase-1 in glioma, we performed additional computational analyses assessing its expression in relation to multiple immune escape associated signatures (Figure 9). Consistently, patients exhibiting high Regnase-1 expression displayed strong positive associations with transcriptional signatures indicative of T-cell exhaustion, senescence, anergy, and enhanced T-cell evasion (Figure 9A). Moreover, GSEA revealed significant enrichment of pathways involved in tolerance induction, negative regulation of immune responses, and apoptosis of inflammatory immune cells, consistent with a diminished antitumor immune response (Figure 9B,C; Supplementary Figure S9). Collectively, these results underscore that Regnase-1 may be linked to the establishment and maintenance of an immunosuppressive tumor microenvironment, thereby facilitating immune escape and promoting glioma progression.

## 4. Discussion

The tumor microenvironment in glioblastoma (GBM), the most aggressive form of glioma, is now recognized as a profoundly immunosuppressive milieu, characterized by a complex interplay between tumor cells and immune components, where regulatory signals actively promote tumor growth, immune evasion, and impaired pro-inflammatory responses [57,58,59,60].

In contrast to their success in many solid tumors, current immune checkpoint inhibitors have yielded limited therapeutic benefit in glioma [13,61]. Clinically, large phase III trials evaluating PD-1 blockade with nivolumab, namely the CheckMate 143 (recurrent glioblastoma, nivolumab monotherapy vs. bevacizumab), CheckMate 498 (newly diagnosed MGMT-unmethylated, nivolumab + radiotherapy vs. temozolomide + radiotherapy), and CheckMate 548 (newly diagnosed MGMT-methylated, nivolumab + radiotherapy + temozolomide vs. standard care), failed to demonstrate improved survival among patients with newly diagnosed or recurrent glioblastoma [62,63,64].

Importantly, glioma resistance involves multiple overlapping mechanisms that extend beyond conventional cell-surface immune checkpoints such as PD-1/PD-L1 [13]. Primary and adaptive resistance often reflects compensatory upregulation of alternative immunosuppressive pathways, such as TIM-3 and LAG-3, with growing evidence indicating that post-transcriptional gene regulation constitutes a critical layer governing inflammatory and immune signaling within the tumor microenvironment [65,66,67]. A key example of this regulatory circuit is provided by endoribonucleases of the Regnase family, which selectively degrade mRNAs encoding pro-inflammatory cytokines and immune mediators, limiting excessive immune activation and preserving physiological homeostasis [20]. When dysregulated, this system can contribute to immune dysfunction and facilitate tumor progression. Among these, Regnase-1 serves as a critical intracellular negative regulator of inflammation, operating through mechanisms fundamentally distinct from cell-surface immune checkpoint-driven regulation [35]. These findings establish a rationale for elucidating the clinical and prognostic relevance of Regnase-1 and its associated immunological signature in glioma.

Our analysis of fresh biopsies from our in-house cohort of 40 invasive glioma patients revealed significant upregulation of Regnase-1 mRNA in tumors exhibiting aggressive features. Specifically, Regnase-1 expression was markedly higher in IDH-wildtype compared with IDH-mutant gliomas, increased with tumor grade, and was most elevated in glioblastoma. No significant association was observed with age or sex. Collectively, these results suggest that Regnase-1 overexpression is associated with aggressive tumor phenotypes, and with features of glioma malignancy. Given the limited cohort size, validation in larger datasets such as TCGA and CGGA is warranted to further assess this hypothesis.

Large-scale analyses of the TCGA and CGGA cohorts corroborated our initial findings from fresh biopsies, demonstrating that elevated Regnase-1 transcript levels are strongly associated with aggressive clinicopathological features indicating poor prognosis in glioma patients.

Higher Regnase-1 expression consistently correlated with advanced tumor grade, with the strongest elevation observed in GBM, as well as with IDH-wildtype status, absence of 1p/19q codeletion, and older age (≥50 years) across both datasets. Minor sex differences were observed, with no association detected in the TCGA cohort and higher expression in males within the CGGA cohort, likely reflecting cohort-specific variation. Kaplan–Meier analyses in both cohorts showed that elevated Regnase-1 expression was linked to significantly shorter overall survival, highlighting its robust prognostic value. Multivariate Cox regression analyses further evaluated this association after adjustment for established clinicopathological and molecular variables. While Regnase-1 showed only a non-significant trend toward increased mortality in the TCGA cohort, it emerged as an independent adverse prognostic factor in the CGGA cohort. This divergence does not undermine the robustness of Regnase-1 as a prognostic biomarker; rather, it reflects systematic differences in cohort architecture. The CGGA dataset is substantially larger (*n* = 959) and enriched for grade IV tumors (37.5%), providing greater power to detect modest hazard ratios. In contrast, the TCGA cohort (*n* = 672, 23.2% grade IV) has a lower event rate, and its multivariate model includes age, IDH status, and tumor grade; among these, IDH status and grade are tightly correlated with Regnase-1 expression and absorb much of the variance that would otherwise be attributed to it. The univariate Kaplan–Meier analyses in both cohorts (Figure 2G,N) nevertheless demonstrate robust and highly significant survival differences (*p* < 0.0001), confirming a consistent biological signal. Moreover, the directionality of the multivariate hazard ratio is concordant across both datasets, aligning with the established literature that cohort-specific demographic, molecular subtype, and treatment-standard variations can modulate the independent prognostic weight of a given biomarker in glioma [68,69,70]. More broadly, systematic reviews have documented that prognostic gene signatures in glioblastoma frequently show variable performance across TCGA and CGGA cohorts, and that differences in cohort selection, clinical covariates, and treatment era can lead to discrepancies in multivariate significance even when univariate associations remain robust [60,71,72]. Taken together, these cross-cohort findings reinforce the conclusion that Regnase-1 overexpression is firmly linked to poor outcome, even if its independent contribution in multivariate models is attenuated in subsets with a lower prevalence of high-grade disease.

Emerging data position Regnase-1 as a context-dependent regulator across cancers. In glioma, Regnase-1 has been implicated in driving tumor aggressiveness through upregulation of vascular endothelial growth factor A (VEGFA) and subsequent activation of the ERK signaling pathway [40]. A similar oncogenic role has been reported in non-small cell lung cancer (NSCLC), where higher Regnase-1 expression correlates with poor prognosis [73]. In stark contrast, Regnase-1 functions as a tumor suppressor in other malignancies. In breast cancer, it induces apoptosis Via degradation of anti-apoptotic transcripts such as Bcl2L1 and RelB, with low expression linked to poor survival. This tumor-suppressive role extends to pancreatic ductal adenocarcinoma (PDAC), where its downregulation promotes MDSC-mediated immune evasion and worse outcomes, and to colorectal cancer (CRC), where low expression associates with aggressive features, poor prognosis, and enhanced IL-17 signaling-driven growth [22,23,74]. Interestingly, in melanoma, higher Regnase-1 expression correlates with improved melanoma-specific survival by inhibiting Akt/mTOR signaling and promoting cell cycle arrest [75]. In neuroblastoma, overexpression of Regnase-1 reduces proliferation and viability through transcriptome and microRNA modulation [76]. These contrasting roles reveal the dual role of Regnase-1. It suppresses tumors in epithelial-derived cancers through inflammation control and apoptosis, yet promotes tumors Via microenvironment remodeling and angiogenesis. This duality underscores its potential as a biomarker and therapeutic target tailored to specific tumor contexts.

To further assess the hypothesis linking elevated Regnase-1 expression to glioma aggressiveness, we performed gene set enrichment analysis using predefined gene sets relevant to glioma biology. We observed strong positive enrichment for pathways associated with tumor progression, including invasion, angiogenesis, hypoxia, epithelial–mesenchymal transition (EMT), and mesenchymal/endothelial glioblastoma features. Key signaling cascades, such as IL6–JAK–STAT3, TNF-α/NF-κB, TGF-β, and PI3K/AKT/mTOR, were also significantly enriched. Interestingly, we also noted enrichment of mRNA catabolism-related signatures, including RNA nuclease activity, endonuclease activity, stem-loop binding, and miRNA catabolism, pointing to a potential role for Regnase-1’s endoribonuclease function in active transcriptome reprogramming that favors malignancy. These findings extend known mechanisms in glioma, where Regnase-1 upregulates VEGFA and activates ERK signaling, offering a plausible explanation for the observed enrichments in hypoxia, EMT, and mesenchymal/endothelial phenotypes in our Regnase-1-high tumors [40].

The composition of tumor-infiltrating immune cells holds substantial prognostic significance due to their critical influence on tumor progression, metastatic potential, and therapeutic response. Within the tumor microenvironment, heterogeneous immune cell populations exert either tumor-promoting or tumor-suppressive effects, depending on their phenotype, functional state, and spatial distribution [77,78]. Considering the central role of immune-microenvironment interactions in glioma progression, we next evaluated the immunological relevance of Regnase-1 by examining immune-related features and pathways associated with Regnase-1-high. Notably, these tumors displayed an immune enriched microenvironment, characterized by increased immune and stromal scores, reduced tumor purity, and a significant enrichment of pathways involved in regulating innate and adaptive immunity, suggesting a possible role for Regnase-1 in modulating the immune landscape of glioma. Indeed, Regnase-1 is expressed on multiple immune cell types, including tumor infiltrating immune cells [37,38,79]. Regnase-1-high tumors also displayed reduced purity, an independent adverse prognostic factor in glioma. Low tumor purity correlates with shorter overall survival, earlier disease recurrence, enhanced invasive and metastatic potential, epithelial–mesenchymal transition (EMT), upregulation of inhibitory immune checkpoints and immunosuppressive chemokines, and increased infiltration by protumoral immune populations, particularly M2-polarized macrophages and regulatory T cells (Tregs) [79,80,81].

To assess Regnase-1 expression in the glioma microenvironment at single-cell resolution, we analyzed the Glioma_GSE163108_10X and Glioma_GSE131928_10X datasets from the Tumor Immune Single-cell Hub 2 (TISCH2) database. This analysis revealed that Regnase-1 is expressed not only in infiltrating immune cells but also in malignant glioma cells. Within tumor cells, Regnase-1 retains its endoribonuclease activity and has been shown to promote tumor progression; Wang et al. reported that it upregulates VEGFA and activates the ERK signaling pathway, thereby enhancing proliferation, migration, and angiogenesis [40]. To determine which immune populations may be driving the Regnase-1-associated immune landscape, we next examined its expression across immune lineages. The TISCH2 datasets consistently showed preferential Regnase-1 expression within immune cells, with the highest levels in regulatory T cells and exhausted CD8^+^ T cells. This dual expression pattern, together with the functional mechanisms characterized in other malignancies, where Regnase-1 directly degrades effector-promoting mRNAs in T cells and drives tumor-cell proliferation, led us to hypothesize that Regnase-1 could simultaneously contribute to glioma-cell aggressiveness and to the sculpting of an immunosuppressive microenvironment by influencing the function of key immune regulatory and effector populations.

Further analysis of the tumor-infiltrating immune cell landscape revealed that, relative to the Regnase-1 low phenotype, tumor microenvironments with high Regnase-1 expression contained a greater abundance of protumorigenic cells, including M0 and M2 macrophages, Treg subpopulations, and exhausted CD8^+^ T lymphocytes. It is worth noting that the LM22 signature matrix was originally derived from peripheral blood leukocytes, and its use may therefore underestimate the full complexity of tissue-resident and tumor-specific immune phenotypes in glioma, including microglia [54,82]. Nevertheless, the strong concordance between the different deconvolution methods, together with independent single-cell RNA-seq data, indicates that the principal immunosuppressive signatures we detected are robust. The development of a glioma-tailored reference matrix remains a worthwhile direction for future refinement.

Consistent with our observation, Regnase-1 has been shown to drive M2 macrophage polarization by suppressing NF-κB and JNK/c-Myc signaling during tissue repair and inflammation resolution, suggesting a link to the immune landscape reported here [83]. Similarly, the enrichment of exhausted CD8^+^ T cells and reduction in effector CD8^+^ T cells in Regnase-1-high tumors reflect its restraint on CD8^+^ responses.

Indeed, Regnase-1 deletion has been reported to reprogram CD8^+^ T cells into long-lived effectors with superior persistence, mitochondrial fitness, and anti-tumor efficacy by targeting BATF and promoting a precursor exhausted state that limits terminal exhaustion [37]. Furthermore, the regulatory interplay between Regnase-1 and the RNA-binding protein Roquin has emerged as a critical axis in immune regulation. Their physical interaction, when disrupted, enhances effector function and tumor accumulation in tumor-specific T cells [79,84]. Despite their interaction, they govern distinct immunological processes. Roquin deficiency primarily impairs Tfh cell differentiation and humoral immunity, whereas Regnase-1 deficiency preferentially drives Th17-mediated inflammatory responses [79]. Moreover, Regnase-1 knockdown in a Roquin-deficient background further enhances Th17 differentiation, indicating that the two proteins operate through non-overlapping mechanisms [85]. Collectively, these observations position Regnase-1 as a potential immunomodulatory target for T cell activity in glioma, where its manipulation could reshape the tumor immune landscape toward an antitumor phenotype. Beyond conventional T cell subsets, Regnase-1-high tumors displayed a reduced abundance of antitumor effector populations, including activated NK, B cells, and effector memory CD8^+^ T cells. In NK cells, Regnase-1 loss potentiates OCT2/IκBζ–NF-κB-mediated transcription of IFN-γ, thereby reinforcing antitumor immunity [38]. In B cells, Regnase-1 deletion promotes proliferation and class-switch recombination, culminating in severe immunopathology and underscoring its essential function in restraining B cell-driven autoimmunity [86].

The composition of the cellular compartment is a well-recognized determinant of cancer prognosis. However, it is ultimately the molecular mediators secreted by immunosuppressive populations, or those driving their recruitment and polarization toward a protumor, tolerogenic state, that orchestrate the tumor microenvironment and signal aggressive disease [87]. Accordingly, we next investigated the relationship between our gene of interest and a panel of inhibitory immune checkpoints [88].

Notably, Regnase-1 expression correlated positively with multiple emerging immune checkpoints, suggesting a possible cooperative or synergistic role for these molecules within the glioma microenvironment. To resolve cell-type-specific immune profiles not captured by bulk transcriptomics, we employed intracellular digital cytometry. Our findings revealed that elevated Regnase-1 expression is associated with coordinated functional suppression across multiple immune compartments. CD8^+^ T cells displayed reduced cytotoxic mediators, including IFNG, GZMA/B, and PRF1, alongside upregulation of exhaustion markers such as PDCD1, CTLA4, LAG3, HAVCR2, and TIGIT; a profile consistent with terminal exhaustion. Regulatory T cells exhibited enhanced suppressive capacity through increased expression of CTLA4, PDCD1, LAG3, HAVCR2, and NT5E. NK cells showed elevated inhibitory receptors, including TIGIT and KLRC1, pointing to impaired cytotoxicity. Myeloid cells, particularly M2-like macrophages, expressed high levels of ARG1 and IL10, reinforcing an immunosuppressive, tumor-supportive milieu. These patterns are supported by functional studies across multiple experimental models. Wei et al. [37] demonstrated that Regnase-1 deletion in CD8^+^ T cells reduces exhaustion markers, including PD-1, TIM-3, CTLA4, and LAG-3, while enhancing effector function, with markedly higher IFN-γ and granzyme B production upon restimulation, leading to superior antitumor efficacy in melanoma and leukemia models. Extending this to adoptive cell therapy, Zheng et al. [89] showed Regnase-1 deficiency in CAR-T cells promotes TCF-1+ precursor states and reduces exhaustion markers such as PD-1, LAG3, TIGIT, HAVCR2 in subsets, thereby enhancing persistence and antitumor activity against B-ALL. Similarly, Mai et al. [79] found combined disruption of Regnase-1 and Roquin-1 unleashes CAR-T antitumor function without strongly increasing exhaustion markers like PD-1, TIM-3, LAG3, or TIGIT in certain models, though effects remain context-dependent.

Prompted by these findings, we asked whether Regnase-1 promotes immune escape in glioma by orchestrating broader transcriptional programs. Our results further underscore the immunosuppressive role of Regnase-1 in immune evasion, emphasizing its potential as a valuable biomarker for immune dysregulation in glioma. Indeed, high Regnase-1 expression is associated with markedly increased signatures of T-cell exhaustion, senescence, anergy, and evasion, together with enrichment of pathways that negatively regulate immune responses, are linked to tolerance, and are associated with apoptosis of inflammatory immune cells, further highlighting the strong association between Regnase-1 and impaired antitumor T-cell function in the glioma microenvironment. This aligns closely with key studies demonstrating Regnase-1’s function as a major negative regulator of antitumor CD8^+^ T-cell responses; its deletion reprograms T cells to avoid exhaustion, enhance persistence, and boost effector function in tumors, as shown in adoptive T-cell therapy models [37]. Similarly, Regnase-1 promotes T-cell exhaustion by limiting the formation of TCF-1+ precursor exhausted T cells; its deficiency increases TCF-1 expression, reduces exhaustion markers, and improves CAR-T cell expansion, memory-like differentiation, and antitumor efficacy [89]. These mechanistic insights from T-cell engineering contexts strongly support how elevated Regnase-1 in glioma contributes to the observed T-cell dysfunction signatures, reinforcing its role in sustaining immunosuppression.

In light of the above mechanistic insights, which align with our own data in the glioma microenvironment, we propose the hypothesis that, within the immune compartment, Regnase-1 functions as a driver of CD8^+^ T-cell dysfunction, not a secondary consequence of the immunosuppressive milieu, and that its elevated expression in glioma actively sustains, rather than simply reflects, the exhausted state. These findings thus position Regnase-1 as a compelling candidate for direct experimental validation in glioma-specific models, aimed at definitively confirming its causative role in CD8^+^ T-cell exhaustion within the glioma microenvironment.

The next logical question is whether this causal role can be exploited therapeutically. The broader translational landscape already establishes Regnase-1 as a druggable and clinically actionable molecule. The PIN-domain catalytic center of Regnase-1 has been solved at atomic resolution (2.75 Å), revealing a well-defined catalytic pocket whose dimer interface is essential for ribonuclease activity and provides a structural blueprint for small-molecule inhibitor design [90]. An independent therapeutic modality, antisense oligonucleotide-mediated modulation of Regnase-1 expression, has entered preclinical development, with the rationale that selective inhibition in immune effector cells can potentiate antitumor immunity [91]. Most compellingly, the adoptive transfer of autologous tumor-infiltrating lymphocytes in which both SOCS1 and Regnase-1 have been inactivated by CRISPR/Cas9 gene editing is currently under evaluation in an active Phase 1/2 clinical trial for advanced solid tumors (NCT06598371) [92]. Preclinical data supporting this trial show that dual inactivation of Regnase-1 and SOCS1 enhances CAR-T cell proliferation, tumor infiltration, and in vivo antitumor efficacy against solid-tumor xenografts, even at substantially reduced cell doses [93]. Regnase-1-deficient CAR T cells targeting B7-H3 further extend this principle, as they not only display improved intrinsic effector function but also reshape the tumor microenvironment toward a pro-inflammatory state with increased infiltration of endogenous IFN-γ-producing T cells and NK cells and reduced M2 macrophages [94]. Given the deeply exhausted and immunosuppressed phenotype we observe in Regnase-1-high gliomas, these convergent translational advances indicate that Regnase-1 is a realistic and high-priority candidate for glioma-directed immunotherapy, whether approached through genetically engineered adoptive cell products already entering the clinic or through future small molecule or ASO-based ribonuclease inhibitors.

Beyond the molecular and clinical dimensions, the Regnase-1-associated immune landscape we describe can also be interpreted through the lens of cancer ecology. An increasing body of work conceptualizes tumors as complex ecosystems in which malignant cells and diverse immune populations engage in continuous interactions that mirror ecological relationships such as predation, competition, and mutualism [95]. Viewed through this perspective, the immunosuppressive microenvironment we find associated with high Regnase-1 expression, characterized by an abundance of regulatory T cells, M2-polarized macrophages, and exhausted CD8^+^ T cells, can be regarded as an ecological state in which tumor-supportive mutualisms dominate over immune predation. Within this framework, Regnase-1 may act as a niche-constructing factor that stabilizes the immunosuppressive ecosystem, thereby facilitating tumor progression and immune evasion. Accordingly, therapeutic strategies aimed at disrupting Regnase-1 could be regarded as a form of “ecosystem engineering” that remodels the glioma microenvironment toward an immune-competent state [96].

We acknowledge that retrospective transcriptomic cohorts carry inherent limitations related to cohort-specific patient composition, clinical heterogeneity, and evolving treatment standards across eras; these factors likely contribute to the inter-cohort variability we observe and underscore the need for prospective validation. Moreover, the transcriptomic associations we report, encompassing clinicopathological, oncogenic and immunosuppressive signatures, call for confirmation at the protein level, together with targeted genetic approaches such as CRISPR-Cas9-mediated manipulation of Regnase-1 in glioma models, to provide the definitive functional validation these findings now motivate.

Overall, our investigation positions Regnase-1 as a key candidate regulator of glioma pathophysiology, strongly associated with immune evasion and with the potential to influence immunotherapeutic resistance in this aggressive malignancy.

## 5. Conclusions

This study provides the first comprehensive analysis of the immunological relevance of Regnase-1 in human glioma, demonstrating that its elevated expression is strongly associated with aggressive clinicopathological features, poor survival, and a profoundly immunosuppressive tumor microenvironment. Despite limitations, including the modest size of our in-house cohort, our findings establish Regnase-1 as a promising prognostic biomarker and therapeutic target. Future functional and preclinical studies targeting Regnase-1 in glioma are warranted to elucidate the mechanisms driving the immunosuppressive TME and to evaluate ribonuclease-targeted strategies for enhancing the efficacy of immunotherapy in this aggressive malignancy.

## Figures and Tables

**Table 1:** Primer sequences used for real-time PCR analysis.

Gene	Forward Sequence (5′→3′)	Reverse Sequence (5′→3′)
β-actin	5′-GAGATGGCCACGGCTGCTT-3′	5′-GCCACAGGACTCCATGCCCA-3′
Regnase-1	5′-TTCCTGCGTAAGAAGCCACT-3′	5′-AATCGGCACTTGATCCCATA-3′

**Table 2:** Clinicopathological parameters of the in-house cohort.

Clinicopathological Parameters	In House Cases (%) (*n* = 40)	*p*-Value
Age		
<50	35 (87.50%)	0.3370
≥50	5 (12.50%)	
Sex		
Male	22 (55%)	0.5999
Female	18 (45%)	
Grade		
Grade I	13 (32.5%)	0.0005
Grade II	9 (22.5%)	
Grade III	6 (15%)	
Grade IV	12 (30%)	
IDH status		
Wildtype	5 (12.5%)	0.0173
Mutant	6 (15%)	
Missing Data	29 (72.5%)	
Histological Subtype		
Astrocytomas	18 (45%)	0.0002
Oligoastrocytomas	3 (7.5%)	
Ependymomas	7 (17.5%)	
Glioblastomas	12 (30%)	

**Table 3:** Clinicopathological parameters of TCGA cohort.

Clinicopathological Parameters	TCGA Cases (%) (*n* = 672)	*p*-Value
Age		
<50	384 (57.14%)	<0.0001
≥50	286 (42.56%)	
Missing Data	2 (0.30%)	
Sex		
Male	385 (57.29%)	0.2803
Female	285 (42.41%)	
Missing Data	2 (0.30%)	
Grade		
Grade II	248 (36.90%)	<0.0001
Grade III	264 (39.29%)	
Grade IV	156 (23.21%)	
Missing Data	4 (0.60%)	
1p/19q Status		
Codeletion	167 (24.85%)	<0.0001
Non Codeletion	254 (37.80%)	
Missing Data	251 (37.35%)	
IDH status		
Wildtype	202 (30.06%)	<0.0001
Mutant	421 (62.65%)	
Missing Data	49 (7.29%)	
Histological Subtype		
Astrocytomas	194 (28.87%)	<0.0001
Oligoastrocytomas	130 (19.35%)	
Oligodendrogliomas	191 (28.42%)	
Glioblastomas	156 (23.21%)	
Missing Data	1 (0.15%)	
Radiation therapy		
No	198 (29.46%)	<0.0001
Yes	379 (56.40%)	
Missing Data	95 (14.14%)	

**Table 4:** Clinicopathological parameters of the CGGA cohort.

Clinicopathological Parameters	CGGA Cases (%) (*n* = 959)	*p*-Value
Age		
<50	684 (71.32%)	<0.0001
≥50	274 (28.57%)	
Missing Data	1 (0.10%)	
Sex		
Male	562 (58.62%)	0.0043
Female	397 (41.38%)	
Grade		
Grade II	279 (29.10%)	<0.0001
Grade III	315 (32.84%)	
Grade IV	360 (37.54%)	
Missing Data	5 (0.52%)	
1p/19q Status		
Codeletion	212 (22.11%)	<0.0001
Non Codeletion	728 (75.81%)	
Missing Data	19 (1.98%)	
IDH status		
Wildtype	401 (41.81%)	<0.0001
Mutant	506 (52.76%)	
Missing Data	52 (5.42%)	
Histological Subtype		
Astrocytomas	373 (38.90%)	<0.0001
Oligoastrocytomas	21 (2.19%)	
Oligodendrogliomas	200 (20.86%)	
Glioblastomas	360 (37.56%)	
Missing Data	5 (0.52%)	
MGMTp methylation status		
Methylated	449 (46.82%)	0.0002
Un-methylated	358 (37.33%)	
Missing Data	152 (15.85%)	
Chemotherapy		
No	260 (27.11%)	0.0485
Yes	636 (66.32%)	
Missing Data	63 (6.57%)	
Radiation therapy		
No	192 (20.02%)	0.9729
Yes	708 (73.83%)	
Missing Data	59 (6.15%)	

## Data Availability

Data generated from Moroccan glioma patients are not publicly available due to ethical and privacy restrictions but are available from the corresponding author upon reasonable request.

## Supplementary Materials

The following supporting information can be downloaded at: https://www.mdpi.com/article/10.3390/cancers18101658/s1, Supplementary Figure S1: PCA and Permanova analysis during Batch correction processing in CGGA cohort. (A) Before Batch correction (B) After Batch correction. Supplementary Figure S2: Patterns of Covariate Missingness Before and After Imputation in TCGA and CGGA cohorts. Supplementary Figure S3: Enrichment analysis plots illustrate significant enriched gene sets in the Regnase-1-high phenotype, highlighting pathways and biological processes relevant to tumor progression, immune modulation, and post-transcriptional RNA regulation in the CGGA cohort. Supplementary Figure S4: Enrichment analysis plots illustrate significant enrichment of key immune-related biological processes in Regnase-1-high tumors from the CGGA cohort. Supplementary Figure S5: Violin plot illustrating the distribution of Regnase-1 in different populations of immune, malignant and stromal cells for the two datasets: Glioma_GSE163108_10X and Glioma_GSE131928_10X. Supplementary Figure S6: Regnase-1 expression correlated with immune cell infiltration in the TCGA and CGGA cohorts, as determined by Spearman’s rank correlation coefficient. Supplementary Figure S7: Analysis of tumor immune and stromal cell infiltration relative to Regnase-1 expression in TCGA and CGGA glioma cohorts using TIMER and xCell. Supplementary Figure S8: Regnase-1 expression levels were positively correlated with inhibitory immune checkpoints in the TCGA and CGGA cohorts using Spearman’s rank correlation coefficient. Supplementary Figure S9: Enrichment analysis plots illustrate significant enrichment of immunosuppressive and tumor escape pathways in Regnase-1-high patients from the CGGA cohort. Supplementary Table S1: Normalized enrichment scores (NES), nominal *p*-values, and FDR q-values for all gene sets significantly enriched in Regnase-1-high gliomas (*p* < 0.05, FDR q < 0.25). Supplementary Table S2: Gene signatures assessed during Gene Set Enrichment analysis of TCGA and CGGA cohorts.

## Abbreviations

The following abbreviations are used in this manuscript:

ADORA2A	Adenosine A2A receptor
ARG1	Arginase 1
B7-H3	B7 homolog 3 (CD276)
B7-H4	B7 homolog 4 (VTCN1)
BTLA	B and T lymphocyte attenuator
CAFs	Cancer-associated fibroblasts
CD160	Cluster of Differentiation 160
CD28	Cluster of Differentiation 28
CD47	Cluster of Differentiation 47
CD69	Cluster of Differentiation 69
CGGA	Chinese Glioma Genome Atlas
CIBERSORT	Cell-type Identification By Estimating Relative Subsets Of RNA Transcripts
CSF1	Colony-stimulating factor 1
CTLA-4	Cytotoxic T-lymphocyte-associated protein 4
ESTIMATE	Estimation of STromal and Immune cells in MAlignant Tumors using Expression data
GBM	Glioblastoma
GSEA	Gene Set Enrichment Analysis
GZMA	Granzyme A
GZMB	Granzyme B
HAVCR2	Hepatitis A virus cellular receptor 2 (TIM-3)
IDO1	Indoleamine 2,3-dioxygenase 1
IDH	Isocitrate dehydrogenase
IFNγ	Interferon gamma
iTreg	Induced regulatory T cell
KLRC1	Killer cell lectin-like receptor C1 (NKG2A)
LAG-3	Lymphocyte activation gene 3
LGALS9	Galectin 9
MCPIP1	Monocyte Chemotactic Protein-1-Induced Protein 1
MAIT	Mucosal-associated invariant T cell
MDSCs	Myeloid-derived suppressor cells
mRNA	Messenger RNA
NFκBIZ	Nuclear factor kappa B inhibitor zeta
NK	Natural killer cell
NKT	Natural killer T cell
NR2F6	Nuclear receptor subfamily 2 group F member 6
nTreg	Natural regulatory T cell
NT5E	5′-Nucleotidase ecto (CD73)
PD-1	Programmed cell death protein 1
PD-L1	Programmed death-ligand 1
PD-L2	Programmed death-ligand 2
PRF1	Perforin 1
RT-PCR	Reverse transcription polymerase chain reaction
TCGA	The Cancer Genome Atlas
TAMs	Tumor-associated macrophages
TIGIT	T cell immunoreceptor with Ig and ITIM domains
TIM-3	T cell immunoglobulin and mucin-domain containing-3
TISCH2	Tumor Immune Single-cell Hub 2
Tr1	Type 1 regulatory T cell
Treg/Tregs	Regulatory T cell(s)
VISTA	V-domain immunoglobulin suppressor of T-cell activation (VSIR)
γδ T cells	Gamma delta T lymphocytes
β-actin	Beta-actin
