# Supplementary material for: High Regnase-1 Expression Is Associated with an Immunosuppressive Tumor Microenvironment and Aggressive Features in Glioma Patients
Source: Cancers (Basel). 2026 May 20;18(10):1658. doi: 10.3390/cancers18101658 (PMC13204960; doi:10.3390/cancers18101658)
Supplement: Supplementary file 1 [file cancers-18-01658-s001.zip › cancers-4252987_Supplementary Figure S5.pdf]

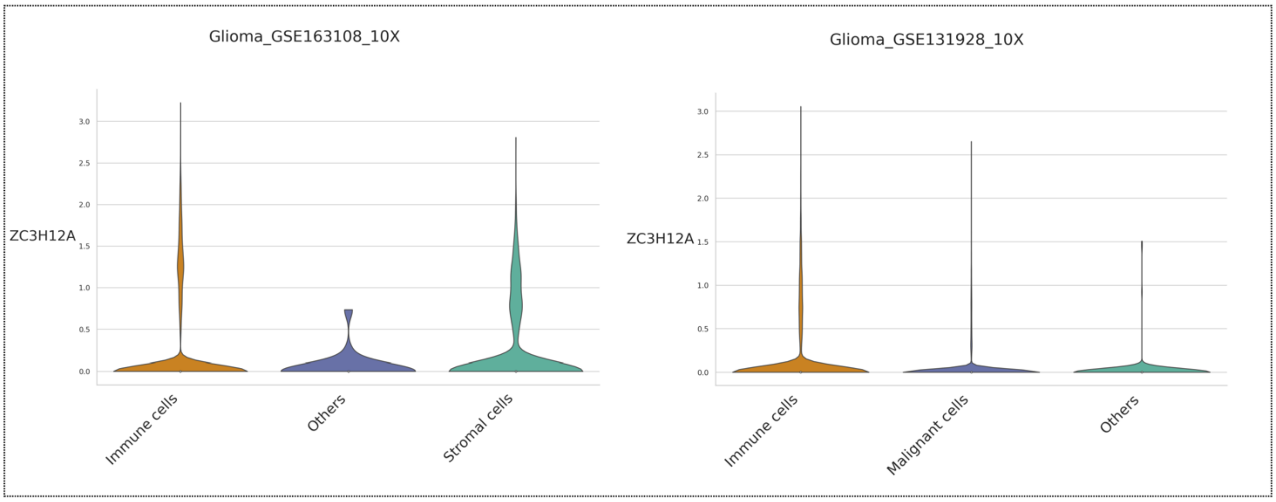

Supplementary Figure S5. Violin plot illustrating the distribution of Regnase-1 in different populations of immune, malignant and stromal cells for the two datasets: Glioma_GSE163108_10X and Glioma_GSE131928_10X.
