# Supplementary material for: High Regnase-1 Expression Is Associated with an Immunosuppressive Tumor Microenvironment and Aggressive Features in Glioma Patients
Source: Cancers (Basel). 2026 May 20;18(10):1658. doi: 10.3390/cancers18101658 (PMC13204960; doi:10.3390/cancers18101658)
Supplement: Supplementary file 1 [file cancers-18-01658-s001.zip › cancers-4252987_Supplementary Figure S7.pdf]

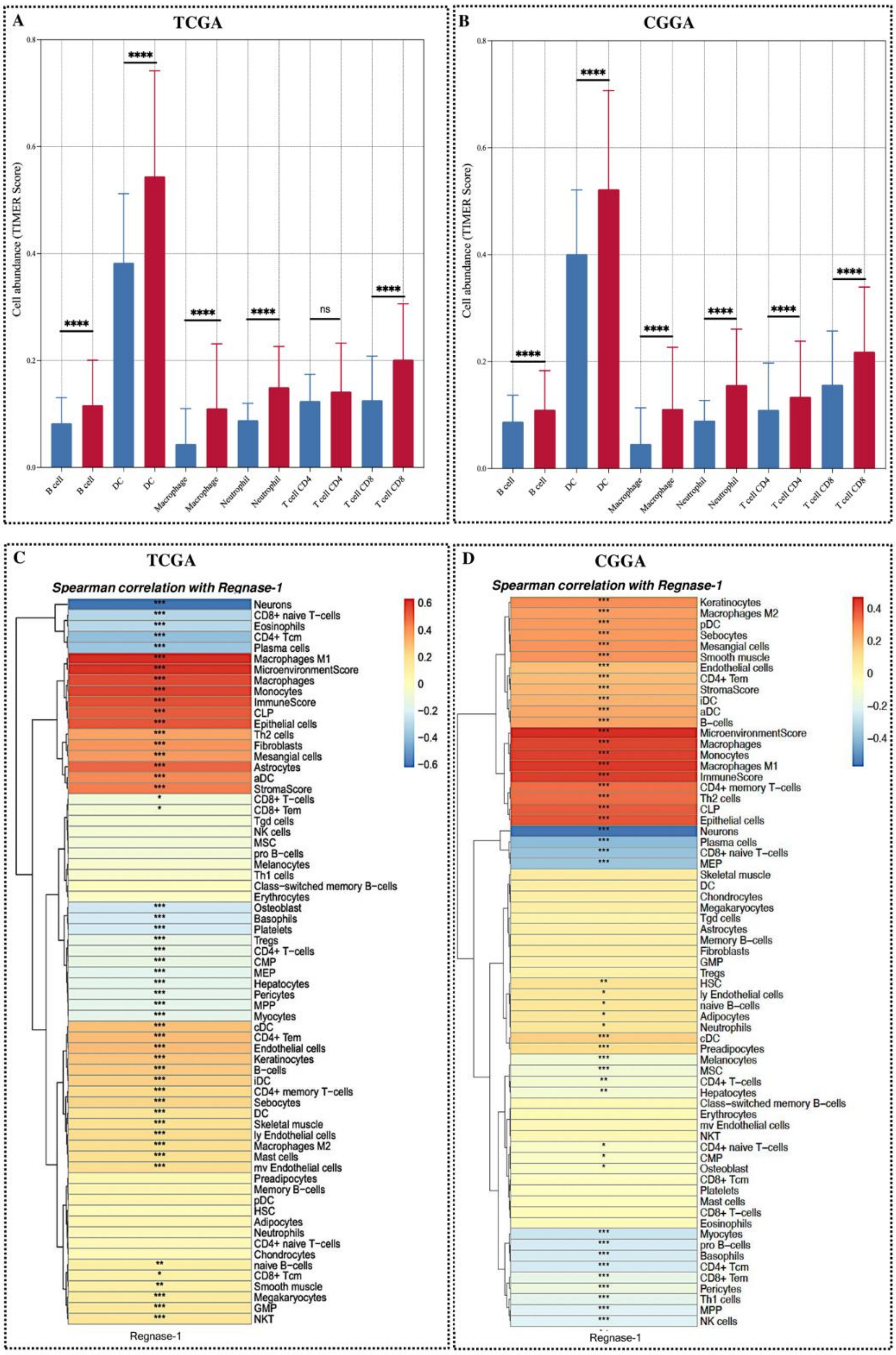

Supplementary Figure S7. Analysis of tumor immune and stromal cell infiltration relative to Regnase-1 expression in TCGA and CGGA glioma cohorts using TIMER and xCell. (A, B) Immune infiltration analysis using TIMER in TCGA and CGGA cohorts. Bar plots represent the estimated fractions of immune cell populations in tumors stratified by Regnase-1 expression (high vs. low). Red bars represent high Regnase-1 phenotype while the blue ones represent low Regnase-1 phenotype. **(C, D)** Correlation between Regnase-1 expression and xCell-derived scores in gliomas. The heatmap displays correlations between Regnase-1 expression and immune, stromal, and tumor microenvironment related cell scores across TCGA and CGGA cohorts. Color intensity represents the strength and direction of correlation (red, positive correlation; blue, negative correlation). Statistical significance is indicated as follows: * $p < 0.05$; ** $p < 0.01$; *** $p < 0.001$; **** $p < 0.0001$; ns, not significant. . Significance was defined as Benjamini-Hochberg corrected FDR < 0.05.
