# Supplementary material for: High Regnase-1 Expression Is Associated with an Immunosuppressive Tumor Microenvironment and Aggressive Features in Glioma Patients
Source: Cancers (Basel). 2026 May 20;18(10):1658. doi: 10.3390/cancers18101658 (PMC13204960; doi:10.3390/cancers18101658)
Supplement: Supplementary file 1 [file cancers-18-01658-s001.zip › cancers-4252987_Supplementary Figure S4.pdf]

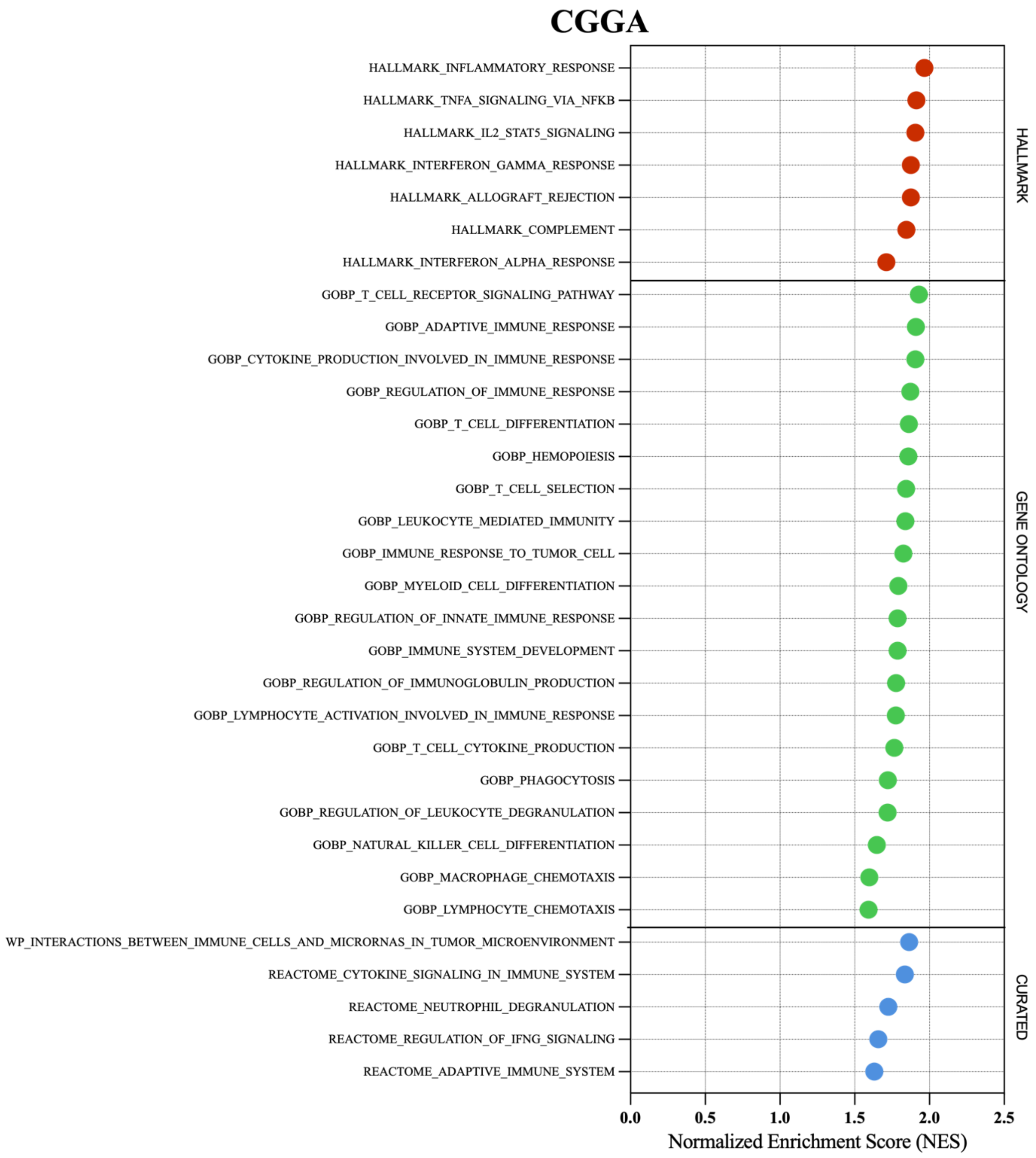

Supplementary Figure S4. Enrichment analysis plots illustrate significant enrichment of key immune-related biological processes in Regnase-1-high tumors from the CGGA cohort. Gene sets were considered significantly enriched at nominal $p < 0.05$ and FDR q-value < 0.25.
