# Supplementary material for: High Regnase-1 Expression Is Associated with an Immunosuppressive Tumor Microenvironment and Aggressive Features in Glioma Patients
Source: Cancers (Basel). 2026 May 20;18(10):1658. doi: 10.3390/cancers18101658 (PMC13204960; doi:10.3390/cancers18101658)
Supplement: Supplementary file 1 [file cancers-18-01658-s001.zip › cancers-4252987_Supplementary Figure S1.pdf]

A

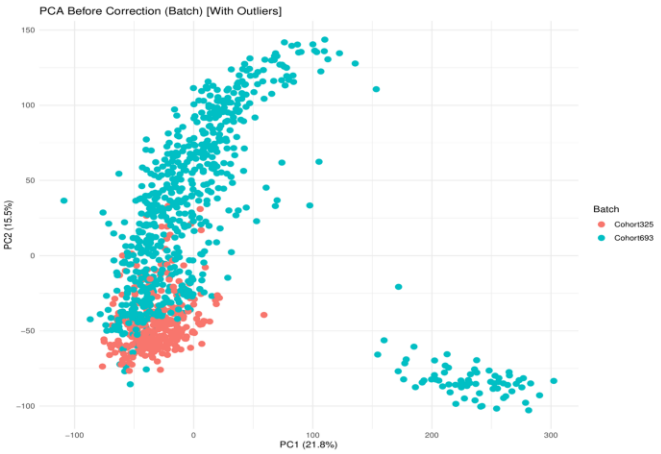

	R^2	pvalue
**Batch**	0,10105	0,001
**1p19q codeletion**	0,05587	0,001
**Age**	0,00431	0,002
**Gender**	0,05907	0,001
**Grade**	0,08758	0,001
**Histology**	0,08758	0,001
**IDH Status**	0,1302	0,001

B

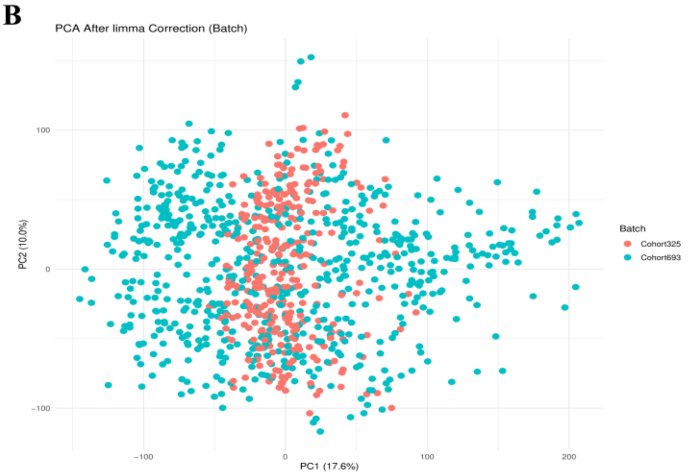

	R^2	pvalue
**Batch**	0	1
**1p19q codeletion**	0,06089	0,001
**Age**	0,01341	0,001
**Gender**	0,00425	0,001
**Grade**	0,06074	0,001
**Histology**	0,08731	0,001
**IDH Status**	0,12252	0,001

Supplementary Figure S1. PCA and Permanova analysis during Batch correction processing in CGGA cohort. **(A)** Before Batch correction. **(B)** After Batch correction.
R^2: residual effect; p-value > 0.05 means statistically non-significant. Significance was defined as Benjamini-Hochberg corrected FDR < 0.05.
