# Supplementary material for: High Regnase-1 Expression Is Associated with an Immunosuppressive Tumor Microenvironment and Aggressive Features in Glioma Patients
Source: Cancers (Basel). 2026 May 20;18(10):1658. doi: 10.3390/cancers18101658 (PMC13204960; doi:10.3390/cancers18101658)
Supplement: Supplementary file 1 [file cancers-18-01658-s001.zip › cancers-4252987_Supplementary Figure S2.pdf]

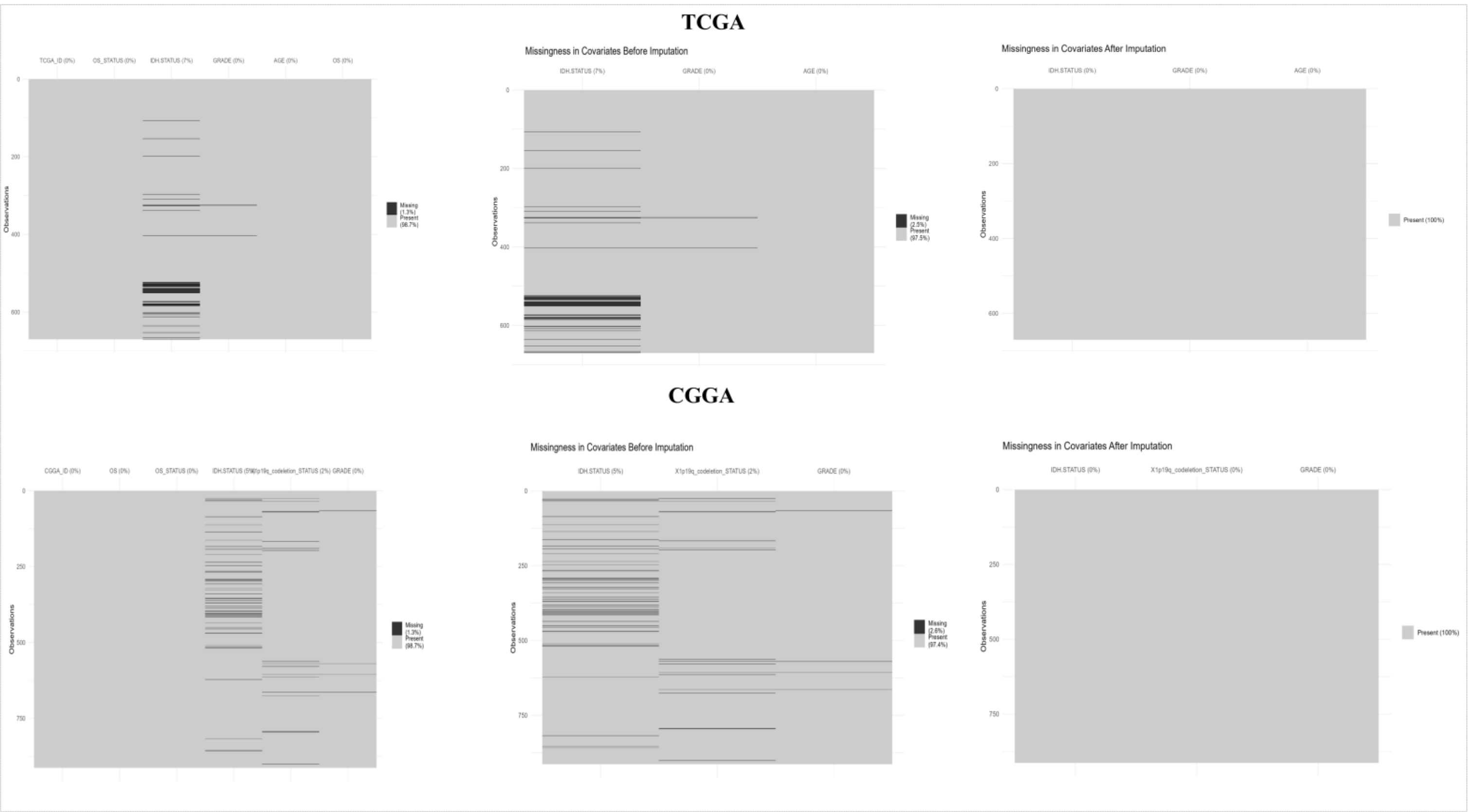

Supplementary Figure S2. Patterns of Covariate Missingness Before and After Imputation in TCGA and CGGA cohorts.
