# Supplementary material for: High Regnase-1 Expression Is Associated with an Immunosuppressive Tumor Microenvironment and Aggressive Features in Glioma Patients
Source: Cancers (Basel). 2026 May 20;18(10):1658. doi: 10.3390/cancers18101658 (PMC13204960; doi:10.3390/cancers18101658)
Supplement: Supplementary file 1 [file cancers-4252987_Supplementary Figure S6.pdf › cancers-4252987_Supplementary Figure S6.pdf]

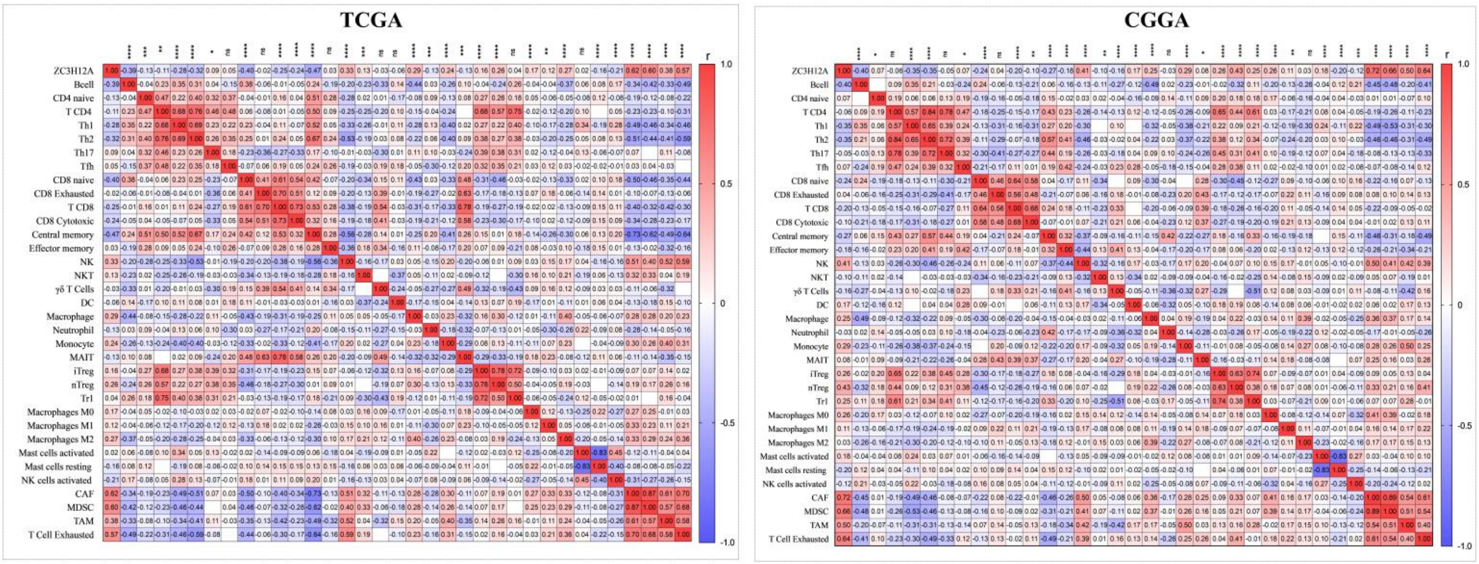

Supplementary Figure S6. Regnase-1 expression correlated with immune cell infiltration in the TCGA and CGGA cohorts, as determined by Spearman's rank correlation coefficient. * $p < 0.05$, ** $p < 0.01$, *** $p < 0.001$, **** $p < 0.0001$, and 'ns' for no significant difference. Significance was defined as Benjamini-Hochberg corrected FDR < 0.05.
